# HED-ID: a federated expert system for interpretable and resource-adaptive intrusion detection on edge devices

**DOI:** 10.1038/s41598-026-61336-6

**Published:** 2026-09-15

**Authors:** Kushboo Nasir, Sahar K. Badri, Asad Khattak, Daniyal M. Alghazzawi, Mohammed Yahya Alghamdi, Mona Alkhozae, Abeer Almakky, Rania M. Alhazmi, Muhammad Zubair Asghar

**Affiliations:** 1 Gomal Research Institute of Computing (GRIC), Faculty of Computing, Gomal University, D.I.Khan (KP), Pakistan; 2 Department of Information Systems, Faculty of Computing and Information Technology, King Abdulaziz University, Jeddah, Saudi Arabia; 3 College of Technological Innovation, Zayed University, P.O. Box 144534, Abu Dhabi, UAE; 4 Department of Computer Science, Faculty of Computing and Information, Al-Baha University, Al-Baha, Saudi Arabia; 5 Department of Information Technology, Faculty of Computing and Information Technology, King Abdulaziz University, Jeddah, Saudi Arabia

**Keywords:** Expert systems, Intrusion detection, Edge computing, Federated learning, Resource-adaptive optimization, Convergent metaheuristics, Explainable AI, Engineering, Mathematics and computing

## Abstract

The proliferation of edge computing in industrial and IoT networks necessitates expert systems that support accurate, interpretable, and resource-efficient intrusion detection under strict privacy and computational constraints. This paper presents **HED-ID**, a fully integrated **federated expert system** designed for real-time, edge-oriented anomaly detection. The system combines: (i) a stacked bidirectional GRU (BiGRU) architecture with hybrid temporal–spatial attention for sequential and feature-level anomaly modeling, (ii) a resource-adaptive Grey Wolf Optimizer (A-GWO) that modulates exploration–exploitation behavior based on real-time CPU utilization and Bayesian epistemic uncertainty, with formal convergence proof under bounded resource shifts (Appendix A), (iii) federated SHAP, which aggregates local explanations using weighted averaging to produce consistent global attributions without sharing raw data, (iv) a multi-objective fitness function that simultaneously considers accuracy, model complexity, and predictive uncertainty, and (v) SHAP-guided iterative pruning for reducing computational overhead while preserving model fidelity. Empirical evaluation on CICIDS-2017, UNSW-NB15, ToN-IoT, and a noisy-edge variant of ToN-IoT (10–20% controlled packet loss and jitter) demonstrates 95.6% accuracy in cloud-like conditions and 93.8% on Raspberry Pi 4 (4 GB RAM) before pruning, with inference latency maintained within 18–22 ms. Post-pruning, memory usage decreases from 92 to 115 MB to 78–96 MB, with ≤ 1.1% accuracy degradation. To support deployment, Docker orchestration templates, pruning threshold configurations, and Grafana-based monitoring are provided—reducing retraining interventions from 11 to 4 per year in a simulated 50-device edge environment.

**Supplementary Information:**

The online version contains supplementary material available at https://doi.org/10.1038/s41598-026-61336-6.

## Introduction

### Background and significance

Edge computing’s rapid adoptiondelete demands intrusion detection systems capable of delivering high accuracy and transparency under severe resource constraints and strict privacy requirements. Although deep learning models achieve strong performance on CICIDS-2017, UNSW-NB15, and ToN-IoT^1–3^, most rely on static architectures that fail to adapt to runtime CPU/memory fluctuations and lack privacy-preserving distributed explainability—limitations that violate regulations such as GDPR.

HED-ID closes this gap by introducing a federated expert system that integrates resource-adaptive metaheuristic optimization with formal convergence guarantees, hybrid attention mechanisms, federated SHAP explainability, and SHAP-guided pruning—simultaneously ensuring detection effectiveness, interpretability, efficiency, and operational manageability on constrained edge hardware.

### Motivation and research gap

Deep learning–based intrusion detection systems (IDS) demonstrate strong performance on benchmark datasets; however, their practical deployment on edge devices remains challenging. Predominant limitations include static model configurations that do not adapt to runtime CPU and memory variability, and explainability mechanisms that are either local-only or centrally aggregated, thereby constraining privacy-preserving interpretability in federated environments. Recent studies clearly exemplify this fragmentation. FLEX-IDS^1^ incorporates federated learning with local SHAP explanations but relies on fixed hyperparameters and omits pruning strategies. A metaheuristic-optimized federated IDS^2^ leverages particle-swarm optimization for DDoS detection, yet decouples optimization from resource-awareness and restricts explainability to centralized post-aggregation. Similarly, a spiking neural network–based federated IDS^3^ prioritizes energy efficiency but employs fixed optimization without convergence guarantees and provides only local explanations.

Collectively, these approaches advance privacy and edge compatibility, but none unifies (i) resource-adaptive metaheuristic optimization supported by a formal convergence analysis, (ii) federated SHAP for globally consistent and privacy-preserving explanations, (iii) SHAP-guided pruning for efficiency-aware model refinement, and (iv) explicit containerized deployment guidance for operational feasibility. This gap underscores the need for an integrated framework that simultaneously ensures detection robustness under resource fluctuations, interpretable decision support, convergence stability, and manageable edge deployment.

To address these challenges, HED-ID introduces a federated expert system that combines hybrid attention-based modeling with provably convergent resource-adaptive optimization, federated SHAP explainability, and SHAP-guided pruning. The framework is specifically designed to sustain detection accuracy, computational efficiency, and globally coherent interpretability in resource-constrained edge settings.

### Research questions

#### RQ1

How effectively does hybrid temporal–spatial attention improve anomaly detection performance in edge-based network traffic compared to standard stacked bidirectional GRU (S-BiGRU) models without attention?

#### RQ2

To what extent does the resource-adaptive metaheuristic optimizer—with formal convergence guarantees under bounded edge resource variation—enhance stability and solution quality on resource-constrained devices?

#### RQ3

How consistently can federated SHAP explanations be aggregated across distributed edge nodes while maintaining local data privacy and minimizing cross-node attribution drift?

#### RQ4

What accuracy–latency–memory trade-offs are achieved through SHAP-guided pruning under constrained edge deployment conditions?

#### RQ5

How do the proposed deployment guidelines (container orchestration, pruning-threshold tuning, and Grafana-based monitoring) reduce operational management overhead in realistic edge network environments?

### Research contributions

This study presents HED-ID, a federated expert system for interpretable and resource-adaptive intrusion detection on edge devices. The key contributions are:

1. A hybrid attention-based architecture (stacked BiGRU with temporal–spatial attention) achieving high detection accuracy on edge hardware with memory below 115 MB and inference latency of 18–22 ms.
2. A resource-adaptive Grey Wolf Optimizer (A-GWO) that dynamically balances exploration–exploitation using real-time CPU utilization and Bayesian epistemic uncertainty, with formal convergence proof under bounded resource variations (Appendix A).
3. Federated SHAP with weighted averaging of local explanations to deliver globally consistent, privacy-preserving interpretability across edge nodes.
4. SHAP-guided iterative pruning that reduces memory from 92 to 115 MB to 78–96 MB with ≤ 1.1% accuracy degradation.
5. Comprehensive deployment guidelines (Docker orchestration, pruning-threshold algorithm, Grafana monitoring) that reduce yearly retraining from 11 to 4 events in a 50-device simulation.

**Fig. 1:**
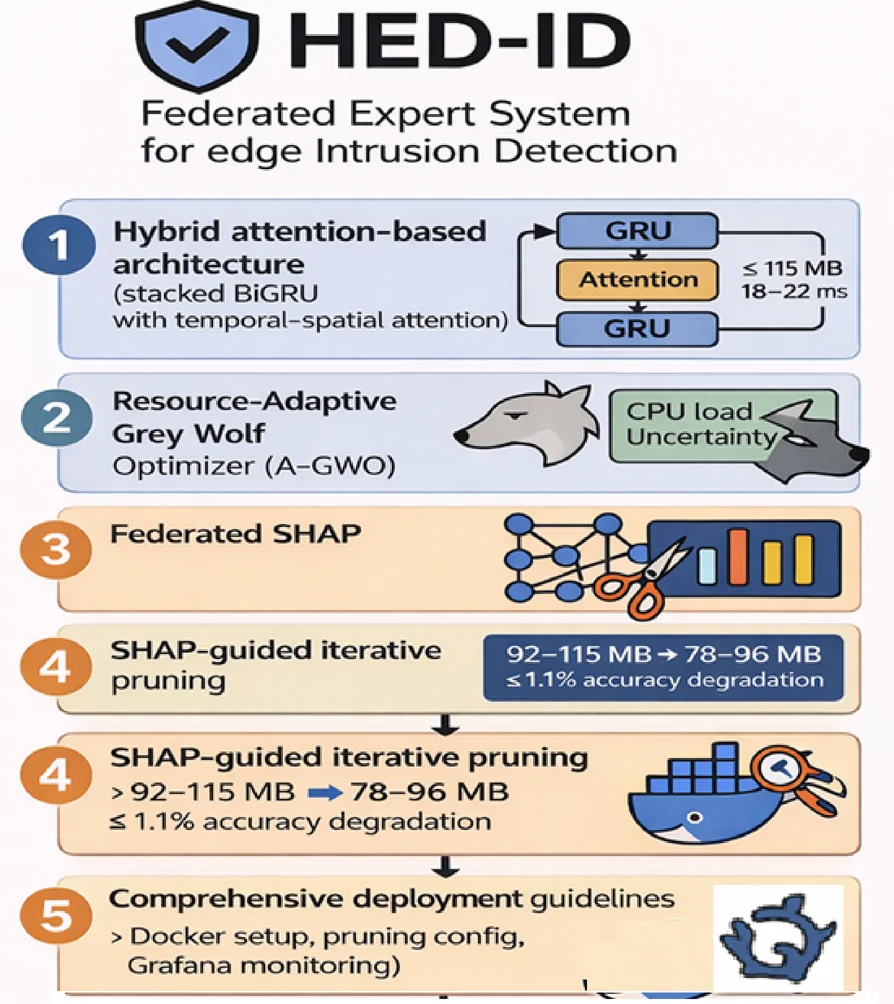
Core contributions of the proposed HED-ID federated expert system for interpretable and resource-adaptive edge intrusion detection.

All components are integrated and openly available in supplementary material, enabling immediate deployment on resource-constrained edge devices. Figure 1 illustrates the core contributions of the proposed HED-ID federated expert system for edge intrusion detection.

### Paper organization

The paper is organized as follows. Section “Literature review” reviews baseline studies and identifies the research gap. Section “Methodology” details the HED-ID federated expert system, covering hybrid attention modeling, resource-adaptive optimizer with convergence proof, federated SHAP, pruning, and deployment guidelines. Section “Experimental setup” presents the experimental setup. Section “Results and discussion” presents results on standard and noisy-edge datasets, ablation studies, and operational performance on Raspberry Pi 4. Section “Conclusions and future work” concludes the study and discusses future work.

## Literature review

### Introduction

Research on intrusion detection systems for edge environments has increasingly incorporated federated learning and explainability techniques. To maintain focus and avoid dispersion, this section briefly summarizes broader methodological trends before concentrating on studies that directly compete with the proposed HED-ID framework. This section reviews representative advances in deep learning, optimization, and explainable AI, followed by a focused analysis of three baseline works most closely aligned with federated and edge-based IDS. The analysis identifies gaps in integration that motivate HED-ID’s federated expert system.

### Representative advances in edge IDS

Deep learning models have been widely explored for edge intrusion detection, often emphasizing lightweight and hybrid designs. Shimin et al.^4^ propose a hybrid deep learning–federated framework combining autoencoders, CNNs, and BiLSTM, achieving 96.2% accuracy. An explainable AI approach for cross-layer intrusion detection^5^ employs SHAP for interpretable anomaly detection, reporting F1-scores of 0.94–0.96.

Metaheuristic optimization has similarly been applied to enhance IDS performance. A hybrid deep learning metaheuristic model^6^ reports 98.5% accuracy and 0.97 F1-score, while metaheuristic-based feature selection approaches^7,8^ achieve up to 97.8% accuracy. Federated explainable AI studies^9,10^ highlight the value of SHAP/LIME integration for interpretability, with typical accuracies above 94%.

While these studies demonstrate progress in accuracy, optimization, or interpretability, they address these dimensions independently rather than as an integrated resource-adaptive and federated explainability framework.

### Baseline studies (direct competitors)

Given the objectives of HED-ID, we concentrate on three recent studies that combine federated learning with edge-oriented IDS design.

FLEX-IDS^1^ implements federated learning with local SHAP explanations, achieving 98.05% accuracy. However, the framework relies on fixed hyperparameters, lacks runtime resource adaptation, provides only local explanations, and omits pruning or deployment protocols.

A metaheuristic-optimized federated IDS^2^ applies particle-swarm optimization for DDoS detection, reaching 97.3% accuracy. The optimization process is not resource-aware, explainability remains centralized, and no federated explanation distribution or compression mechanisms are included.

A spiking neural network–based federated IDS^3^ prioritizes energy efficiency with 95.2% accuracy. Nevertheless, optimization parameters remain static, convergence stability under resource variability is not analyzed, and explanations are limited to local scope.

**Research Gap Summary**: Across these direct competitors^1–3^, federated learning and edge deployment are addressed only partially: resource adaptation is not integrated into optimization, explainability is local-only or centralized rather than globally consistent, and efficiency mechanisms (e.g., SHAP-guided pruning) and deployment protocols are typically omitted. This motivates HED-ID as an integrated federated expert system that jointly targets stable edge inference under resource variability, globally coherent interpretability, and deployable efficiency-aware operation.

#### Baseline reporting policy:

The performance values listed for FLEX-IDS^1^, the metaheuristic-optimized federated IDS^2^, and the spiking NN-based federated IDS^3^ in Table 1 are reported from their original publications (i.e., not reproduced in our environment) and are used here to contextualize architectural coverage and missing capabilities (resource adaptation, global explainability, pruning, and deployment support). Because reported results may reflect different dataset splits, client counts, feature sets, and hardware, they are not assumed to be directly comparable to HED-ID. To enable a fair, identical-condition comparison, Table 13A introduces a reproduced baseline (FLEX-IDS^1^ implemented within our experimental pipeline and evaluated on the same datasets and metrics used for HED-ID.

**Table 1:** Comparative Analysis of Related and Baseline Studies.

Study	Year	Federated Learning	Resource Adaptation	Global Explainability	Pruning/Compression	Deployment Guidelines	Convergence Proof	Accuracy (%)	F1-Score
FLEX-IDS^1^	2025	Yes	No	Local only	No	No	No	98.05	0.86
Metaheuristic-optimized federated IDS^2^	2025	Yes	No	Centralized post-aggregation	No	No	No	97.3	N/A
Spiking NN-based federated IDS^3^	2025	Yes	No	Local only	No	No	No	95.2	N/A
Shimin et al^4^.	2025	Yes	No	No	No	No	No	96.2	N/A
Cross-layer XAI IDS^5^	2025	No	No	Local SHAP	No	No	No	N/A	0.94–0.96
Hybrid DL metaheuristic^6^	2025	No	No	No	No	No	No	98.5	0.97
Metaheuristic feature selection^7^	2025	No	No	No	No	No	No	97.8	N/A
XAI in cybersecurity review^8^	2025	Discussed	No	Discussed	No	No	No	N/A	0.92–0.95
ML-IDS with XAI evaluation^9^	2025	No	No	Local SHAP/LIME	No	No	No	94.7	0.93
HED-ID (this work)	2025	Yes	Yes	Federated SHAP	Yes (SHAP-guided)	Yes (Docker/Grafana)	Yes	95.6 (cloud)/93.8 (edge)	0.95 (cloud)/0.93 (edge)

### Comparative analysis

Table 1 summarizes the comparison between representative studies, direct baselines, and HED-ID.

## Methodology

**Fig. 2:**
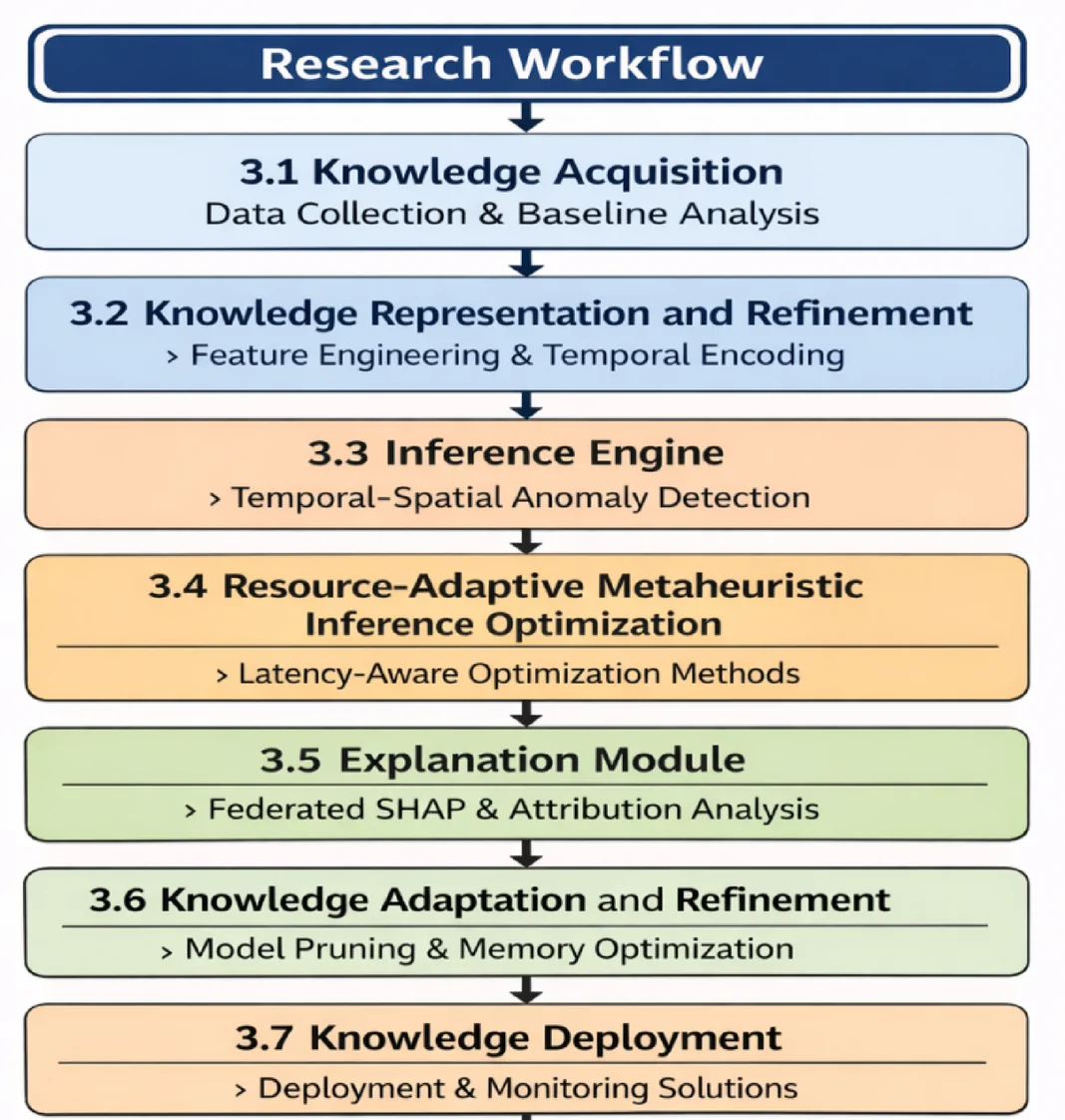
Workflow of the proposed HED-ID framework for edge intrusion detection. **Notation and dimensions**: Let $\:{x}_{t}\in\:{R}^{d}$ denote the feature vector at time step * t*, where * d* is the feature dimension and $\:t\in\:\{1,\dots\:,T\}$ for a sequence length * T*. Scalars are written in normal font, vectors in bold (e.g., **X**), and $\:\parallel\:\cdot\:{\parallel\:}_{2}$ denotes the Euclidean norm.

This section presents the architecture of HED-ID, a federated expert system for edge intrusion detection. Following the classical expert system components—knowledge acquisition, representation, inference, adaptation, and explanation—HED-ID maps learning-based modules to these functions while providing formal guarantees and modular deployment support. Each subsection corresponds to a core component and directly addresses the research questions. The overall workflow of the proposed framework is illustrated in Fig. 2.

For clarity and consistency of mathematical expressions used throughout the methodology, Table 2 summarizes the principal symbols and variables employed in the proposed HED-ID framework.

**Table 2:** Notation summary.

Symbol	Description
$\:{C}_{fusion}\:$	Post-attention fused context vector used for classification and SHAP attribution
$\:{\phi\:}_{j}$	SHAP value representing contribution of feature * j*
$\:{\phi\:}_{j}^{+}$	Positive SHAP value: $\:max({\phi\:}_{j},0)$
$\:{S}_{i}$	Local SHAP attribution vector at node * i*
$\:{S}_{fed}$	Federated (global) SHAP attribution vector
$\:{\omega\:}_{i}$	Node reliability $\:weight\:=\frac{{N}_{i}}{{\phi\:}_{i}+\epsilon\:}$
$\:{N}_{i}$	Number of samples at node$\:\:i$
$\:{\sigma\:}_{i}$	Standard deviation of SHAP values (attribution variability)
$\:{\varDelta\:}_{SHAP}$	Attribution Drift metric (Eq. 15)
$\:{H}^{+}$	Explanation Concentration entropy over positive SHAP values
* d*	Feature dimension

### Knowledge acquisition *(data acquisition and knowledge base construction)*

An expert system begins by acquiring diverse and context-rich data to serve as the knowledge base. HED-ID is evaluated on three public IDS benchmarks—**UNSW-NB15**^11^, **CICIDS-2017**^12^, and **ToN-IoT**^13^—and the proposed **ToN-IoT-EdgeNoise** variant (Sect. “Software stack”). All datasets are harmonized into a unified feature ontology and transformed into flow-based temporal sequences to match the BiGRU–hybrid-attention inference backbone (Sect. “Knowledge representation and refinement for temporal reasoning”).

#### Dataset acquisition (datasets)

The selected benchmarks span enterprise traffic (UNSW-NB15, CICIDS-2017), IoT telemetry (ToN-IoT), and edge-degraded conditions (ToN-IoT-EdgeNoise), enabling evaluation of detection accuracy, robustness, and interpretability under heterogeneous operational constraints. For methodological consistency, datasets are standardized to a shared feature space and encoded into fixed-length flow sequences for temporal modeling (Sect. “Knowledge representation and refinement for temporal reasoning”).

**A. Benchmark Dataset Characteristics**.

Table 3 summarizes the scale, attack diversity, and application domains of the datasets used in this study. The selected benchmarks vary significantly in size and threat composition, providing a realistic testbed for evaluating model generalization and stability under both balanced and heterogeneous traffic distributions.

**Table 3:** Overview of benchmark datasets used in the HED-ID framework.

Dataset	No. of Records	Attack Types	Domain
UNSW-NB15^11^	2.54 M	9 (e.g., DoS, Exploits, Reconnaissance, Generic)	Enterprise network
CICIDS-2017^12^	2.83 M	14 (e.g., Brute Force, Botnet, Infiltration, Heartbleed)	Enterprise traffic
ToN-IoT^13^	22.5 M	6 (e.g., DDoS, Injection, Backdoor, XSS, MITM)	IoT/Smart environments
ToN-IoT-EdgeNoise (this work)	10,000	4 (DDoS, Injection, Reconnaissance, Benign)	Simulated edge-degraded environment

**B. ToN-IoT-EdgeNoise Dataset**.

##### Dataset Motivation:

To evaluate model robustness under realistic edge-network instability, we constructed the ToN-IoT-EdgeNoise dataset by extending the standard ToN-IoT^13^ benchmark with controlled synthetic perturbations. While ToN-IoT captures IoT and cloud-integrated attack scenarios, it does not explicitly model communication degradations typical of constrained edge environments. The proposed dataset therefore introduces noise patterns reflecting delay variability, transmission jitter, and packet loss, enabling systematic assessment of detection stability and explanation consistency under degraded conditions.

**Noise Injection Protocol**: Noise was introduced through an SDN-based emulation framework designed to replicate moderate edge-network impairments. Specifically, three perturbation components were applied: (i) uniform random delay within the range [5–25 ms], simulating scheduling and queuing latency; (ii) Gaussian-distributed jitter modeled as *N*(0,8 ms), representing timing variability; and (iii) Bernoulli packet loss with probability *p* = 0.02, emulating intermittent transmission failures. These parameters approximate practical edge-network fluctuations observed in IoT and wireless deployments, introducing controlled stochastic variability without distorting the underlying attack semantics.

##### Reproducibility:

Noise generation and dataset transformation were implemented using Python-based preprocessing scripts, ensuring deterministic replication through fixed random seeds (seed = 42). All perturbations were applied consistently across training, validation, and test partitions, preserving comparability of robustness evaluations. This design guarantees that experimental variability reflects model behavior rather than stochastic inconsistencies in noise generation.

#### Dataset partitioning

Each dataset is split into **70%/15%/15%** train/validation/test using stratification to preserve class proportions. For **ToN-IoT** and **ToN-IoT-EdgeNoise**, we additionally adopt cross-device evaluation by training on one device group and testing on a disjoint device set to assess domain transfer under heterogeneous sources; for ToN-IoT-EdgeNoise, stratification also preserves noisy vs. clean proportions across splits (Table 4).

**Table 4:** Partitioning strategy for datasets used in this study.

Dataset	Training Set (70%)	Validation Set (15%)	Testing Set (15%)	Special Strategy
UNSW-NB15^11^	Stratified samples of all 9 attack types and normal flows	Balanced validation subset for tuning	Independent stratified test subset	None
CICIDS-2017^12^	Stratified samples of all 14 attack types and normal flows	Balanced validation subset	Independent stratified test subset	None
ToN-IoT^13^	Stratified samples of IoT telemetry	Balanced validation subset	Cross-device testing (trained on one IoT environment, tested on another)	Cross-device validation
ToN-IoT-EdgeNoise (*this work*)	Stratified by attack class and noise-injection status	Balanced stratification including noisy flows	Cross-device and noise-aware testing (with degraded latency, jitter, loss)	Cross-device + noise-aware validation

### Knowledge representation and refinement for temporal reasoning

**Fig. 3:**
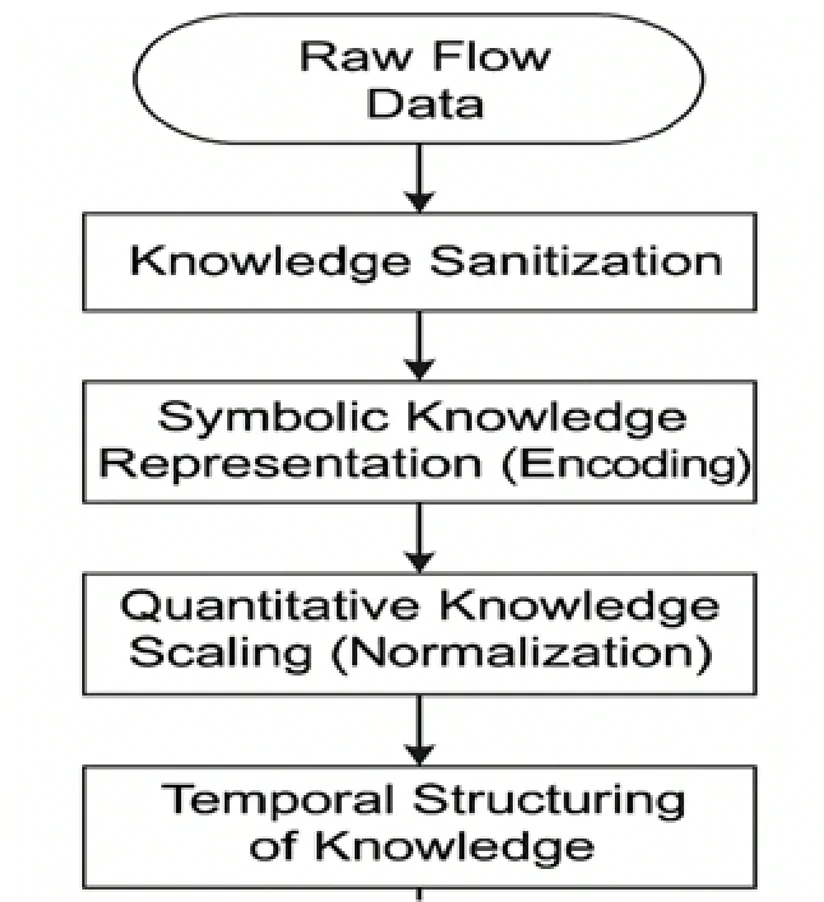
Standardized knowledge refinement pipeline for preparing flow-based intrusion data.

In expert systems, the quality and structure of the knowledge base are central to reliable inference. To ensure consistent inference across datasets, HED-ID applies a unified refinement pipeline comprising (i) sanitization, (ii) schema-aligned symbolic encoding, (iii) normalization, and (iv) temporal sequencing (Fig. 3). This produces the standardized temporal knowledge base consumed by the inference engine (Sect. “Inference engine: temporal–spatial reasoning core for edge intrusion detection”) and explanation module (Sect. “Explanation module: federated SHAP attribution for transparent reasoning”).

#### Knowledge sanitization

Missing numeric entries are imputed using mean substitution, and flows with > 5% missing attributes are removed. Duplicate flows are eliminated using SHA-256 hashing to reduce leakage and overfitting, and extreme outliers in high-variance fields (e.g., $\:src\_bytes,\:duration,\:inter\_arrival\_time$) are moderated using the IQR rule to stabilize downstream scaling^14^.

#### Symbolic knowledge representation (encoding)

Categorical fields (e.g., protocol, service, flags) are one-hot encoded, binary indicators are retained as 0/1, and a consistent ontology-aligned schema is enforced to maintain identical feature dimensionality across datasets—supporting comparable learning and explanation attribution^15^.

#### Quantitative knowledge scaling (normalization)

Continuous features are min–max scaled to [0,1] to prevent magnitude-dominance in optimization and attention weighting^16^::

1 $$\:x^=\frac{x-{x}_{min}}{{x}_{max}-{x}_{min}}$$

where $\:\boldsymbol{x}$ denotes the original feature value, $\:{x}_{min}$ and $\:{x}_{max}$ are the minimum and maximum values of that feature, and $\:\boldsymbol{x}^\in\:\left[\mathrm{0,1}\right]$ is the normalized value.

This ensures balanced feature contributions across heterogeneous flow attributes within the knowledge base.

#### Temporal structuring of knowledge

Flow records are converted to ordered episodes using sliding windows of length T, producing sequences that preserve precursor–outcome dependencies (e.g., $\:reconnaissance\to\:exploit$). The resulting temporal representation forms the input $\:{x}_{1:T}$ to the BiGRU backbone (Sect. “Inference engine: temporal–spatial reasoning core for edge intrusion detection”).

### Inference engine: temporal–spatial reasoning core for edge intrusion detection

The inference engine classifies each flow sequence as benign or malicious by combining stacked BiGRU temporal modeling with hybrid attention over time and features. This design targets **RQ1** by improving discrimination and interpretability without introducing heavy post-fusion layers, preserving edge feasibility^17^.

#### Stacked bidirectional GRU for temporal context modeling

The stacked bidirectional GRU (S-BiGRU) architecture is adopted due to its computational efficiency and its capability to model long-range temporal dependencies in sequential network traffic. Each traffic instance is represented as a temporal sequence:

$$\:{x}_{1},{x}_{2},\dots\:,{x}_{T}, \:{x}_{t}\in\:{R}^{d}$$

**where** $\:\boldsymbol{T}$ denotes the sequence (window) length,$\:\:\boldsymbol{t}\:$is the time-step index, and $\:\boldsymbol{d}$ is the feature dimension.

The forward and backward hidden states are computed as:

2 $$\:{\overrightarrow{h}}_{t}=\:{GRU}_{f}\left({x}_{t},\:{\overrightarrow{h}}_{t-1}\right),\:{\overleftarrow{h}}_{t}={GRU}_{b}({x}_{t},{\overleftarrow{h}}_{t+1)}$$

where $\:{\overrightarrow{h}}_{t}$ and $\:{\overleftarrow{h}}_{t}$ denote the forward and backward hidden-state vectors, respectively, and $\:{\boldsymbol{G}\boldsymbol{R}\boldsymbol{U}}_{\boldsymbol{b}}$ represent the forward and backward GRU cells.

The bidirectional hidden representation is obtained via concatenation:

3 $$\:{h}_{t}=[{\overrightarrow{h}}_{t};\:{\overleftarrow{h}}_{t}]$$

**where** $\:[\cdot\:;\cdot\:]$denotes vector concatenation.

Stacking two BiGRU layers enhances the model’s ability to capture hierarchical temporal structures and evolving attack behaviors (e.g., reconnaissance preceding exploitation). The selection of GRU units over LSTM cells is deliberate, reducing computational overhead and inference latency — a critical requirement for deployment on resource-constrained edge devices.

#### Temporal attention: weighing time steps based on informative content

To better identify time steps that carry significant predictive signals, a temporal attention mechanism is applied. The attention score $\:{\alpha\:}_{t}$ for each hidden state $\:{h}_{t}$ is computed as:

4 $$\:{e}_{t}=\:{v}_{\propto\:}^{T}\mathrm{tanh}\left(\:{W}_{\alpha\:}{h}_{t}+{b}_{\alpha\:}\right)\:\:$$

5 $$\:{\alpha\:}_{t}=\frac{\mathrm{e}\mathrm{x}\mathrm{p}\left({e}_{t}\right)}{{\sum\:}_{i=0}^{T}\mathrm{exp}\left({e}_{i}\right)},\:\:\:\:\:\:\:{c}_{temp}=\:\sum\:_{t=1}^{T}{\alpha\:}_{t}.{h}_{t}$$

where $\:{h}_{t}\in\:{R}^{2u}$ is the BiGRU hidden state at time t (with u units per direction), $\:{\alpha\:}_{t}\in\:\left[\mathrm{0,1}\right]$ is the normalized temporal attention weight, and $\:{W}_{\alpha\:},{b}_{\alpha\:},{v}_{\alpha\:}$ are learnable parameters.

This mechanism helps the model identify subtle precursors to attacks (e.g., short bursts of abnormal port access) that might be diluted in unweighted aggregation. It reflects the inference trace prioritization typically seen in expert reasoning^3^.

#### Spatial (feature-level) attention for focused attribution

Each feature within a time step may contribute differently to classification. Spatial attention is applied to assign weights $\:{\beta\:}_{j}$ to features $\:{X}_{tj}$

6 $$\:{s}_{j}=\:{v}_{\beta\:}^{T}\mathrm{tanh}\left(\:{W}_{\beta\:{x}_{tj}}+{b}_{\beta\:}\right)\:\:\:\:$$

The attended feature vector is:

7 $$\:{\beta\:}_{j}=\frac{\mathrm{e}\mathrm{x}\mathrm{p}\left({s}_{j}\right)}{{\sum\:}_{k=1}^{d}\mathrm{exp}\left({s}_{k}\right)},\:\:\:\:\:\:\:{c}_{spat}=\:\sum\:_{j=1}^{d}{\beta\:}_{j}\cdot{x}_{tj}$$

where $\:{z}_{j}\:$denotes the feature-level embedding associated with feature * j* in the current window (derived from the sequence representation), $\:{\beta\:}_{j}\in\:\left[\mathrm{0,1}\right]$ is the feature attention weight, and $\:{W}_{\beta\:},{b}_{\beta\:},{v}_{\beta\:}$ are learnable parameters.

This layer supports interpretability by making feature contributions explicit and learnable. It is particularly useful when features have skewed importance (e.g., *dst_bytes* and *flow_duration* often dominate during exfiltration attempts).

#### Fusion and classification

The Fusion and Classification stage represents the decision-making core of the inference engine in HED-ID—analogous to the inference and decision output module in traditional expert systems. Temporal and feature attention outputs are concatenated to form the post-attention fused context vector used for classification and explanation^18^.

Let $\:{\mathrm{c}}_{\mathrm{t}\mathrm{e}\mathrm{m}\mathrm{p}}\in\:{\mathrm{R}}^{\mathrm{h}}$ denote the **temporal context vector**, obtained by aggregating BiGRU hidden states weighted by temporal attention coefficients $\:\alpha\:$ (Eqs. 3–4). Let $\:C\_\left(spat\:\right)\in\:{R}^{d}$ represent the **feature-attention vector**, derived from spatial attention weights $\:\boldsymbol{\beta\:}$ (Eqs. 5–6), where $\:\boldsymbol{h}$ is the hidden-state dimension and $\:\boldsymbol{d}$ is the feature dimension.

The two representations are concatenated to form the **post-attention fused context vector**:

8 $$\:{C}_{fusion}=\left[{C}_{temp;\:}{C}_{spat\:}\right]\:\in\:\:{R}^{h+d}$$

**where** $\:[\cdot\:;\cdot\:]$ denotes vector concatenation.

The predicted class probabilities are produced by a lightweight softmax classifier:

9 $$\:{y}^{\hat{}}=Softmax({W}_{c}.{c}_{fusion})+{b}_{c}$$

**where** $\:{\boldsymbol{W}}_{\boldsymbol{c}}\in\:{\boldsymbol{R}}^{\boldsymbol{K}\times\:(\boldsymbol{h}+\boldsymbol{d})}$ is the classification weight matrix, $\:{\boldsymbol{b}}_{\boldsymbol{c}}\in\:{\boldsymbol{R}}^{\boldsymbol{K}}$ is the bias vector, **K** is the number of intrusion classes, and $\:\boldsymbol{y}^{\hat{}}\: \in\:{\boldsymbol{R}}^{\boldsymbol{K}}$ is the predicted probability distribution over classes.

Using $\:{\boldsymbol{C}}_{\boldsymbol{f}\boldsymbol{u}\boldsymbol{s}\boldsymbol{i}\boldsymbol{o}\boldsymbol{n}}$ as the shared prediction–explanation representation ensures consistency between inference and interpretability, aligning the classifier output with federated SHAP attribution (Sect. “Explanation module: federated SHAP attribution for transparent reasoning”).

### Resource-adaptive metaheuristic inference optimization (RQ2)

The inference engine in HED-ID expert system must operate reliably on edge devices subject to CPU, memory, and thermal fluctuations. To maintain stable inference under runtime CPU/memory variability, HED-ID integrates a resource-adaptive Grey Wolf Optimizer (GWO)^19^ that modulates exploration–exploitation using normalized CPU load $\:\rho\:$ and epistemic uncertainty $\:U\left(\theta\:\right)$. The adaptive coefficient replaces the standard GWO control term, and convergence stability is established via Lyapunov analysis (Appendix A).

#### Resource-adaptive coefficient design

In the classical Grey Wolf Optimizer (GWO), the balance between exploration and exploitation is controlled by a linearly decaying coefficient vector A, which decreases monotonically with iterations. While effective in static environments, this formulation does not account for runtime computational variability characteristic of edge deployments.

To incorporate situational awareness, we introduce a resource-adaptive control coefficient, denoted as $\:{A}_{adapt}$, which dynamically modulates the optimizer’s search behavior using real-time CPU utilization and epistemic uncertainty.

The adaptive coefficient is defined as:

10 $$\:{A}_{adapt}=2a\hspace{0.17em}{r}_{1}-a(1-\rho\:)+\beta\:\hspace{0.17em}U\left(\theta\:\right)$$

where $\:\boldsymbol{a}$ is the GWO convergence factor, $\:{r}_{1}$ is a random vector sampled from $\:U\left(\mathrm{0,1}\right)$, $\:\boldsymbol{\rho\:}$ denotes normalized CPU utilization, $\:\boldsymbol{\beta\:}$ controls uncertainty influence, and $\:\boldsymbol{U}\left(\boldsymbol{\theta\:}\right)$ represents epistemic uncertainty of model parameters $\:\theta\:.$.

This formulation enables adaptive search behavior: higher uncertainty or lower CPU load promotes exploration, whereas high CPU utilization induces conservative updates.3.4.3 Integration into Grey Wolf Optimizer.

The designed adaptive coefficient $\:{A}^{\rightarrow}_{adapt}$ directly replaces the conventional $\:A^{\rightarrow}$ term in the GWO’s position update equations, thereby embedding **resource sensitivity into the core optimization loop**.

In standard GWO, the position update for a candidate solution * X(t+1)* is based on the influence of the three best agents ($\:{X}_{\alpha\:},{X}_{\beta\:},{X}_{\delta\:}$)

11 $$\:X\:\left(t+1\right)=\frac{\left({X}_{\alpha\:}\right(t),{X}_{\beta\:}(t),{X}_{\delta\:})}{3}$$

where $\:{X}^{\left(t\right)}\in\:{R}^{m}$ is a candidate solution (optimizer state) at iteration * t* in an * m*-dimensional search space, and $\:\left({X}_{\alpha\:}\right(t),{X}_{\beta\:}(t),{X}_{\delta\:})$ are the three best solutions (leaders) in the current population.

Each of the sub-updates uses:

12 $$\:{X}_{\alpha\:}=\:{X}_{\alpha\:}-{A}_{\alpha\:}.\left|{C}_{\alpha\:}.{X}_{\alpha\:}-X\:\left(t\right)\right|$$

**where**$\:\boldsymbol{X}\left(\boldsymbol{t}\right)$ denotes the current candidate solution vector, $\:{\boldsymbol{X}}_{\boldsymbol{\alpha\:}}\left(\boldsymbol{t}\right)$ is the position of the leading (best) agent, $\:{\boldsymbol{A}}_{\boldsymbol{\alpha\:}}\left(\boldsymbol{t}\right)$ is the exploration–exploitation coefficient, $\:{\boldsymbol{C}}_{\boldsymbol{\alpha\:}}\left(\boldsymbol{t}\right)$ is the stochastic influence vector, *⊙* denotes elementwise multiplication, and $\:\mid\:\cdot\:\mid\:\:$represents the elementwise absolute value.

The stochastic coefficient is defined as:

13 $$\:C=2{r}_{2}\:\mathrm{with}\: \mathrm{r}2\sim\mathrm{U}(0,1)$$

 

**where** $\:{r}_{2}$is a random vector sampled from a uniform distribution.

To incorporate resource awareness, the standard GWO coefficients are replaced with the adaptive formulation:

14 $$\:{A}_{*}=\:{A}_{adapt\:}\left(t\right)\:\:\:\:\:\:\:*\in\:\{\alpha\:,\beta\:,\delta\:\}$$

where $\:{A}_{adapt}\left(t\right)$ is computed using the resource-adaptive control law (Eq. 9), modulated by $\:\rho\:\in\:\left[\mathrm{0,1}\right]$ (normalized CPU load) and $\:U\left(\theta\:\right)$ (epistemic uncertainty).

##### Interpretation:

This substitution enables optimizer dynamics to respond to real-time edge conditions.

- $$\:High\:CPU\:load\:(\rho\:\uparrow\:)\:\to\:\:Aadapt\downarrow\:\:\to\:\:smaller\:updates\:\to\:\:conservative\:search$$.
- $$\:High\:uncertainty\:\left(U\right(\theta\:)\uparrow\:)\:\:\to\:\:Aadapt\uparrow\:\:\to\:\:larger\:updates\:\to\:\:enhanced\:exploration$$.

##### Example:

Under heavy resource utilization (ρ = 0.9), update magnitudes shrink, stabilizing inference. Conversely, when uncertainty rises (U(θ) = 0.25), exploration increases to avoid premature convergence.

Consequently, the metaheuristic optimization module functions as a context-aware adaptive component within the HED-ID expert system, enabling the inference engine to regulate its update dynamics using epistemic uncertainty and real-time resource signals. This mechanism emulates adaptive rule refinement behavior, ensuring stable convergence, resource-sensitive reasoning, and sustained decision reliability under edge constraints.

##### Implementation Details of the Adaptive Optimizer:

To ensure reproducibility and operational clarity, the adaptive Grey Wolf Optimizer (A-GWO) is executed as a local optimization routine at each client node after every local training phase within a federated round. The optimizer operates on a parameter vector that represents inference-related hyperparameters, including the number of GRU units, dropout rate, and attention scaling coefficients.

The normalized CPU utilization is obtained by scaling instantaneous CPU usage to the range [0,1] using min–max normalization over a sliding window of recent measurements. Epistemic uncertainty is computed as the variance of model predictions over 10 Monte Carlo dropout forward passes, followed by normalization to [0,1] to ensure compatibility with the adaptive control formulation.

The adaptive coefficient is recomputed at each iteration of the optimizer and directly modulates the step size of parameter updates. Specifically, when exceeds 0.8, the magnitude of parameter updates is dampened to prevent instability under high resource contention, whereas for below 0.4, exploration is amplified to improve convergence toward optimal configurations.

During each optimization cycle (maximum 20 iterations), candidate solutions are evaluated using the multi-objective fitness function (Sect. 3.4.4), and only the best-performing configuration is retained for subsequent federated aggregation. This design ensures that optimization remains lightweight, resource-aware, and consistent with the constraints of edge deployment while preserving convergence guarantees established in Appendix A.

#### Fitness function

To ensure stable and resource-efficient inference on constrained edge devices, HED-ID employs a multi-objective fitness function within the resource-adaptive optimization loop. This function evaluates candidate inference configurations by jointly considering predictive performance, computational footprint, and model uncertainty.

The scalar fitness is defined as:

14 $$\:\mathrm{F}\:\left(\theta\:\right)\:=\:{\lambda\:}_{1}\:\left(1-Accuracy\right)+\:{\lambda\:}_{2}MemoryUsage+\:{\lambda\:}_{3}\mathrm{E}\mathrm{p}\mathrm{i}\mathrm{s}\mathrm{t}\mathrm{e}\mathrm{m}\mathrm{i}\mathrm{c}\mathrm{U}\mathrm{n}\mathrm{c}\mathrm{e}\mathrm{r}\mathrm{t}\mathrm{a}\mathrm{i}\mathrm{n}\mathrm{t}\mathrm{y}\:$$

Where $\:\theta\:$ denotes the set of tunable inference parameters (e.g., number of GRU units, attention weights, dropout rate), $\:{\lambda\:}_{1},\:{\lambda\:}_{2},{\lambda\:}_{3}$ $\:\in\:$ [0,1] are weight coefficients reflecting the deployment priorities subject to $\:{\lambda\:}_{1}+\:{\lambda\:}_{2}+{\lambda\:}_{3}=1$.

Accuracy is measured on a stratified validation subset, MemoryUsage is recorded during containerized inference on the target edge device, and Epistemic-Uncertainty is estimated via Monte Carlo dropout variance.

Lower values of $\:F\left(\theta\:\right)$ indicate superior trade-offs between detection reliability, computational efficiency, and inference stability under resource variability.

#### Convergence stability via lyapunov analysis

The resource-adaptive GWO must converge reliably despite edge-induced resource fluctuations. Its stability was proved by using Lyapunov stability theory^20^, a standard tool for verifying robustness in control and expert systems.

Let $\:X\left(t\right)$ denote the optimizer state at iteration t (inference parameters) and * X* the optimal configuration minimizing the fitness $\:F\left(\theta\:\right)$. A Lyapunov function is defined as:

15 $$\mathrm{V}\: \mathrm{(t)} = \:{\parallel X\left(t\right)-\:{X}^{*}\parallel}^{2}$$

 

Where $\:{X}^{*}$ denotes an optimal (or near-optimal) solution minimizing $\:F\left(\theta\:\right)$, and $\:\parallel\:\cdot\:{\parallel\:}^{2}$ is the Euclidean norm.

The Lyapunov-based convergence analysis (detailed in Appendix A) shows that, under bounded CPU load and epistemic uncertainty, the resource-adaptive optimizer converges exponentially to a stable, near-optimal inference parameters despite real-time resource fluctuations on devices such as Raspberry Pi 4 and Jetson Nano. This formal stability guarantee aligns adaptive inference with expert-system reasoning principles and directly answers RQ2 by confirming improved stability and solution quality on resource-constrained edge hardware.

##### Summary:

By coupling real-time resource sensing with uncertainty-aware coefficient control and Lyapunov-stable updates, the optimizer delivers robust, low-overhead tuning under edge variability, directly addressing RQ2.

### Explanation module: federated SHAP attribution for transparent reasoning

In expert systems, the explanation module must justify outputs transparently. To ensure methodological completeness and reproducibility, HED-ID implements a fully specified federated SHAP attribution mechanism that defines the SHAP variant, attribution computation level, aggregation strategy, privacy-preserving protocol, communication overhead, and consistency monitoring (RQ3).

#### Local attribution from attention outputs

Each edge node computes SHAP values using DeepSHAP, selected for compatibility with the hybrid BiGRU + temporal–spatial attention architecture. DeepSHAP is employed consistently across all experiments and datasets. SHAP attribution is performed on the post-attention fused representation $\:{C}_{fusion}$ rather than raw input features.

For a prediction $\:y^=f\left({C}_{fusion}\right)$, the SHAP value $\:{\phi\:}_{j}$ quantifies the contribution of feature jjj to the decision, forming a local attribution vector $\:{S}_{i}=[{\phi\:}_{1},{\phi\:}_{2},\dots\:,{\phi\:}_{d}]$ at node * i*. (SHAP is not computed on raw input features in any experiment.)

Across all experiments, DeepSHAP explanations were computed on $\:{C}_{fusion}$, ensuring consistency between prediction and explanation spaces.

#### Privacy-preserving aggregation of SHAP vectors

To preserve data privacy, instance-level SHAP vectors are not shared across nodes. Instead, each node transmits only an aggregated feature-level SHAP summary, thereby preventing disclosure of sample-specific explanations. Specifically, SHAP values computed on the post-attention fused representation $\:f\left({C}_{fusion}\right)$ (Sect. “Local attribution from attention outputs”) are locally averaged before communication. The server then computes the global attribution via reliability-weighted federated averaging:

16 $$\:{S}_{fed}=\frac{{\sum\:}_{i=1}^{N}{\omega\:}_{i}{s}_{i}}{{\sum\:}_{i=1}^{N}{\omega\:}_{i}}$$

where $\:{\boldsymbol{s}}_{\boldsymbol{i}}$ is the local SHAP attribution vector at node * i*, $\:{\boldsymbol{\omega\:}}_{\boldsymbol{i}}$ is the node reliability weight, and $\:\boldsymbol{N}$ is the number of participating nodes.

#### Privacy and security model

Building on the federated SHAP aggregation strategy introduced in Sect. “Privacy-preserving aggregation of SHAP vectors”, the privacy of explanation exchange is ensured through a Secure Aggregation protocol integrated with Homomorphic Encryption (HE). Homomorphic Encryption is adopted to enable mathematically correct aggregation directly in the encrypted domain, eliminating the need to decrypt intermediate node-level explanations.

##### Threat Model:

The aggregation server is assumed **honest-but-curious**, meaning it follows the protocol correctly but may attempt inference. Under HE-based secure aggregation, reconstruction of client-specific explanations is computationally infeasible.

##### Privacy Guarantee:

Raw features, gradients, and instance-level SHAP vectors are never transmitted, ensuring strict preservation of client data confidentiality.

Each node encrypts its aggregated SHAP summary prior to transmission:

17 $$\:{S}_{i}^{\sim}=Enc\left({S}_{i}^{-}\right)$$

Here, $\:{S}_{i}^{-}$denotes the feature-level mean SHAP vector at node i, while $\:{S}_{i}^{\sim}$represents its encrypted form.

Encryption ensures that node-specific attribution statistics remain inaccessible during communication and aggregation.

Global aggregation is performed directly on encrypted vectors:

18 $$\:{S}_{fed}^{\sim}=\frac{\sum\:_{i=1}^{N}{w}_{i}{S}_{i}^{\sim}}{\sum\:_{i=1}^{N}{w}_{i}}$$

Only the final global attribution vector is decrypted:

19 $$\:{S}_{fed}=Dec\left({S}_{fed}^{\sim}\right)$$

This design guarantees that intermediate SHAP summaries and node-level attribution distributions remain confidential.

##### Example:

Even if a node reports a dominant SHAP attribution for $\:dst\_port$, encryption prevents the server from associating this explanation with any specific traffic instance.

#### Communication overhead

Following the privacy-preserving aggregation strategy described in Sect. “Privacy and security model”, the communication overhead of the explanation module remains bounded. **Because only aggregated feature-level SHAP summaries are exchanged**,** rather than instance-level attribution vectors**,** the transmission cost scales linearly with feature dimensionality**:

20 $$\:Overhead\:per\:aggregation=O\left(d\right)\:$$

where * d* denotes the the number of features. This complexity reflects transmission of a single * d-dimensional* vector rather than per-instance explanations.

In practice, each node transmits a single d-dimensional SHAP vector per aggregation cycle. For example, with d = 32 features using 4-byte floating-point encoding, the transmission cost is 128 bytes per node.

To balance interpretability freshness and bandwidth efficiency, federated SHAP aggregation is scheduled periodically:

21 $$\:{f}_{agg}=every\:5\:FL\:rounds\:$$

This aggregation frequency balances explanation freshness and bandwidth efficiency, ensuring negligible overhead relative to model parameter updates.

#### Attribution consistency monitoring

To evaluate the **stability and cross-node coherence** of explanations produced by the federated SHAP aggregation mechanism (Eq. 22), two complementary metrics are monitored: **Attribution Drift** and **Explanation Concentration**.

22 $$\:{\varDelta\:}_{SHAP}=\frac{1}{N}\sum\:_{i=1}^{N}\left|\right|{s}_{i}-{S}_{fed}{\left|\right|}_{2}$$

**where N** denotes the number of participating edge nodes, $\:{\boldsymbol{s}}_{\boldsymbol{i}}$ represents the aggregated local SHAP attribution vector computed at node iii, $\:{\boldsymbol{S}}_{\boldsymbol{f}\boldsymbol{e}\boldsymbol{d}}$ is the global federated SHAP attribution vector obtained via Eq. (14), and $\:\parallel\:\cdot\:{\parallel\:}_{2}$ denotes the Euclidean norm.

##### Interpretation:

Small $\:{\varDelta\:}_{SHAP}$ → explanations are globally consistent, and Large $\:{\varDelta\:}_{SHAP}$ → potential drift or data heterogeneity.

##### Example:

A drift value $\:{\varDelta\:}_{SHAP}$=0.06 indicates minimal deviation between local and aggregated explanations.

##### Explanation Concentration$\:\left({\boldsymbol{H}}^{+}\right)$: 

It is computed as follows.

23 $$\:{H}^{+}=-\sum\:_{j}{p}_{j}\mathrm{l}\mathrm{o}\mathrm{g}\left({p}_{j}\right)$$

24 $$\:{p}_{j}=\frac{\left|{\varphi\:}_{j}^{+}\right|}{{\sum\:}_{k}\left|{\varphi\:}_{k}^{+}\right|}$$

**where** $\:{\boldsymbol{\phi\:}}_{\boldsymbol{j}}$ denotes the SHAP value of feature * j*, $\:{\varphi\:}_{j}^{+}$**=max(**$\:{\boldsymbol{\phi\:}}_{\boldsymbol{j}}$,**0)** represents the positive SHAP contribution, $\:{\boldsymbol{p}}_{\boldsymbol{j}}\:$is the normalized magnitude of the positive attribution for feature * j*, $\:\boldsymbol{d}$ is the feature dimension, and $\:{H}^{+}$ is the explanation concentration entropy.

##### Interpretation:

Lower $\:{H}^{+}$ indicates that a small number of features dominate the explanation, reflecting focused and interpretable reasoning. Higher $\:{H}^{+}$ suggests more diffuse feature contributions.

##### Example:

An explanation dominated by two features yields low entropy (e.g., $\:{H}^{+}$≈0.7), while broadly distributed contributions produce higher entropy (e.g., $\:{H}^{+}$>2).

##### Operational Role:

Together, these metrics enable detection of explanation drift, attribution instability, and retraining requirements, ensuring interpretability robustness under federated and resource-variable edge conditions.

#### Federated SHAP illustration and alignment with RQ3

To illustrate the operation of the proposed explanation module in a realistic expert-system setting, we analyze a representative instance from the ToN-IoT-EdgeNoise dataset. A five-step port-scanning flow characterized by short duration, variable byte counts, and uncommon destination ports is processed independently by three edge nodes. Local SHAP attributions computed on the post-attention fused representation $\:{C}_{fusion}$ are summarized as follows:

- Node A: $$\:src\_bytes\:\left(0.34\right),\:dst\_port\:\left(0.29\right),\:flow\_duration\:\left(0.21\right)$$
- Node B: $$\:src\_bytes\:\left(0.31\right),\:flow\_duration\:\left(0.26\right),\:dst\_port\:\left(0.28\right)$$
- Node C: $$\:dst\_port\:\left(0.30\right),\:src\_bytes\:\left(0.28\right),\:packet\_rate\:\left(0.19\right)$$

Reliability-weighted aggregation (Sect. “Privacy-preserving aggregation of SHAP vectors”, Eq. 14) produces a global explanation dominated by $\:src\_bytes$, $\:dst\_port$, and $\:flow\_duration$, aligning with established domain knowledge of scanning attacks. Explanation stability metrics further indicate: $\:{\varDelta\:}_{SHAP}<0.08$; AND $\:{H}^{+}=low$.

##### Interpretation:

The observed low attribution drift ($\:{\varDelta\:}_{SHAP}$) and reduced entropy ($\:{H}^{+}$) confirm cross-node explanation consistency and focused reasoning, demonstrating robustness of interpretability under federated execution.

##### Example:

Despite node-level variability introduced by packet jitter and noise injection, federated SHAP aggregation consistently attributes anomalous behavior to abnormal destination ports and bursty traffic characteristics.

These observations confirm that HED-ID generates globally coherent, privacy-preserving explanations, directly satisfying the interpretability objective defined in RQ3.

**Fig. 4:**
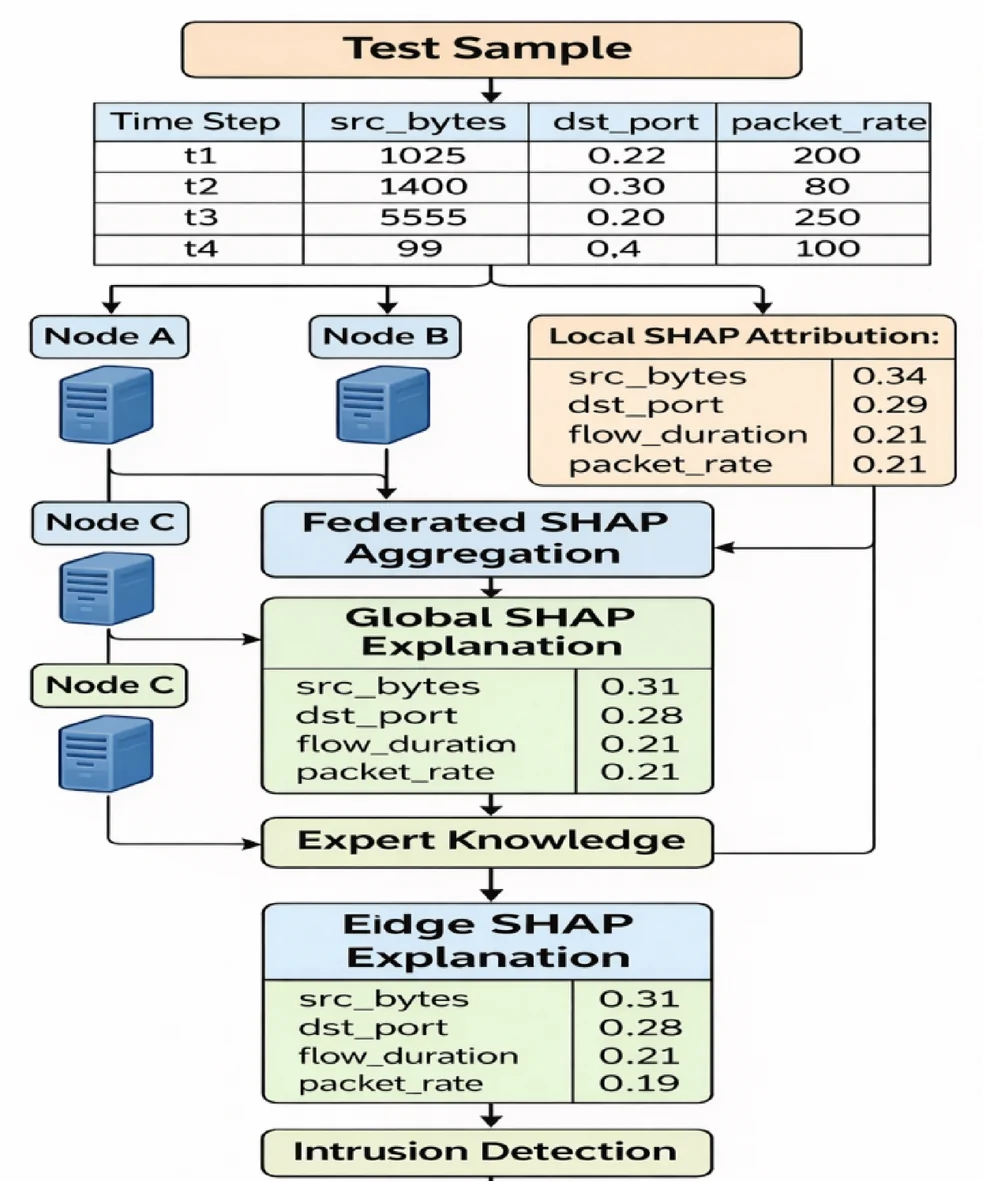
Federated SHAP explanation workflow illustrating local attribution, encrypted aggregation, and globally consistent explanations.

Figure 4 showins local DeepSHAP attribution on $\:{C}_{fusion}$, homomorphically encrypted summary exchange, reliability-weighted aggregation (Eq. 14), and globally consistent feature importance with low $\:{\varDelta\:}_{SHAP}$ and $\:{H}^{+}$.

### Knowledge adaptation and refinement (RQ4)

*(SHAP-Guided Pruning Aligned with RQ4)*

In expert systems, an efficient and interpretable knowledge base is critical on resource-constrained edge devices. HED-ID achieves this via SHAP-guided pruning that removes low-contribution neurons based on their average SHAP magnitude over a sequence:

25 $$\:\frac{1}{T}\sum\:_{t=1}^{T}{\phi\:}_{j}\left(t\right)<\tau\:$$

where $\:{\phi\:}_{j}\left(t\right)$ is the SHAP magnitude of neuron * i* at time$\:\:t$, and τ is a validation-tuned threshold. This removes low-impact components, mirroring rule retraction in classical expert systems.

On the ToN-IoT-EdgeNoise dataset, neurons associated with low-SHAP features (e.g., $\:packet\_interval,\:payload\_entropy$) were pruned while high-impact features ($\:src\_bytes,\:dst\_port,\:flow\_duration$) were retained. Pruning reduced model size by 18–22%, inference latency by ~ 14%, and memory footprint from 92 to 115 MB to 78–96 MB, with accuracy loss ≤ 1.1%.

When applied locally after each federated round, this decentralized, evidence-based refinement mirrors rulebase evolution in classical expert systems, directly satisfying RQ4 by delivering a controlled accuracy–efficiency trade-off while preserving interpretability and modularity on resource-constrained edge devices.

#### Implementation details of SHAP-guided pruning

To operationalize SHAP-guided pruning in a reproducible manner, neuron importance is computed by aggregating SHAP contributions over both temporal steps and validation samples. For each neuron, the pruning score is defined as the average absolute SHAP contribution:

26 $$\:{S}_{j}=\frac{1}{N.T}\sum\:_{n=1}^{N}\:{\sum\:}_{t=1}^{T}|{\phi\:}_{j}^{(n,t)}\mid\:\:\:$$

where denotes the number of validation samples and is the temporal window length. This aggregation ensures that pruning decisions reflect consistent contribution across both time and data instances.

At the end of each federated round, all neurons with are marked for removal. Pruning is applied structurally by eliminating the corresponding weights and connections from the model, followed by a lightweight fine-tuning step (2 epochs on local data) to recover any minor performance degradation.

To prevent excessive pruning and maintain model stability, a safeguard constraint is enforced such that no more than 25% of neurons are removed in a single pruning cycle. Additionally, pruning decisions are applied independently at each client node, ensuring decentralized model adaptation without requiring synchronization of internal model structures.

This iterative pruning–fine-tuning cycle continues across federated rounds, enabling gradual model compression while preserving predictive accuracy and interpretability. The resulting mechanism mirrors rule-base simplification in classical expert systems, where low-utility rules are removed based on accumulated evidence.

### Knowledge deployment (RQ5)

This stage operationalizes HED-ID as containerized, interpretable agents on edge devices and addresses RQ5: how structured deployment reduces operational overhead.

Each node runs in a self-contained Docker container that bundles the hybrid-attention inference engine, federated SHAP module, pruning logic, and resource-adaptive optimizer. Configuration (pruning threshold τ, batch size, CPU limits) is handled via YAML files. Optimal τ is chosen locally via lightweight grid search that jointly minimizes validation loss and latency on the target device (e.g., Raspberry Pi 4):

27 $$\:{\tau\:}^{*}={\mathrm{arg}min}_{\tau\:\in\:\left[\mathrm{0.01,0.05}\right]}({\mathcal{L}}_{val}\left(\tau\:\right)+\lambda\:.Latency\left(\tau\:\right))$$

where $\:\tau\:$ is the SHAP-guided pruning threshold, $\:{\tau\:}^{*}\:$is the selected device-specific threshold, $\:{\mathcal{L}}_{val}\left(\tau\:\right)$ denotes the validation loss obtained after pruning with threshold $\:\tau\:$, $\:Latency\left(\tau\:\right)$ is the measured per-sample inference latency (e.g., in ms) under the same pruning level, and $\:\lambda\:\:\ge\:0$ is a weighting coefficient controlling the accuracy–latency trade-off. The search interval [0.01,0.05] specifies the candidate pruning range explored during deployment tuning.

This balances compactness and reliability on low-power devices by selecting the pruning level that minimizes validation error while penalizing excessive runtime latency.

Containers export key metrics (latency, memory, CPU, SHAP drift) via Prometheus and display them in Grafana. When attribution drift exceeds threshold (EWMA > 0.08), a concept-drift event is triggered for retraining or re-optimization—mirroring lifecycle management in classical expert systems.

#### Illustrative Example:

A smart-factory gateway node (τ = 0.03, 100 MB budget) detects rising SHAP drift from evolving traffic. When drift exceeds δ = 0.1, the monitoring module automatically triggers retraining, minimizing manual intervention while preserving interpretability and stability.

The deployment module thus completes the expert system lifecycle, delivering interpretable, self-adapting reasoning within the resource constraints of edge environments.

**Fig. 5:**
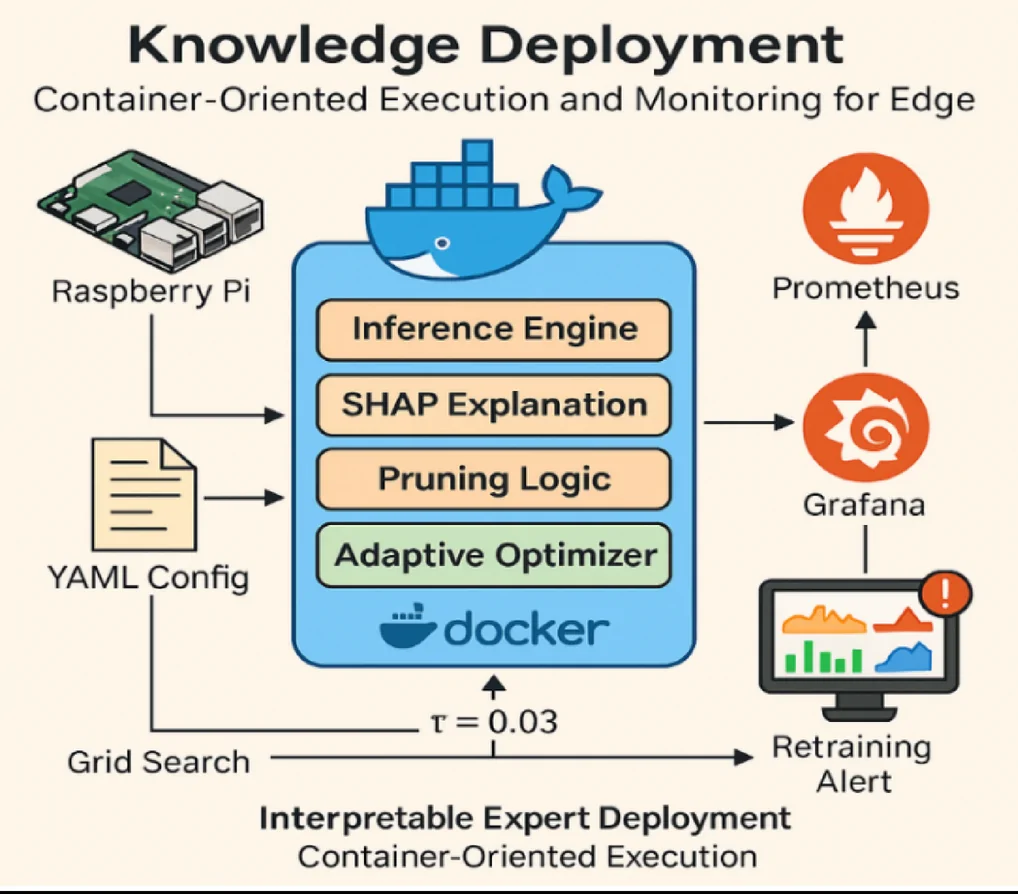
High-level workflow of the knowledge deployment module.

Figure 5 illustrates the high-level workflow of the knowledge deployment module.

**Figure:**
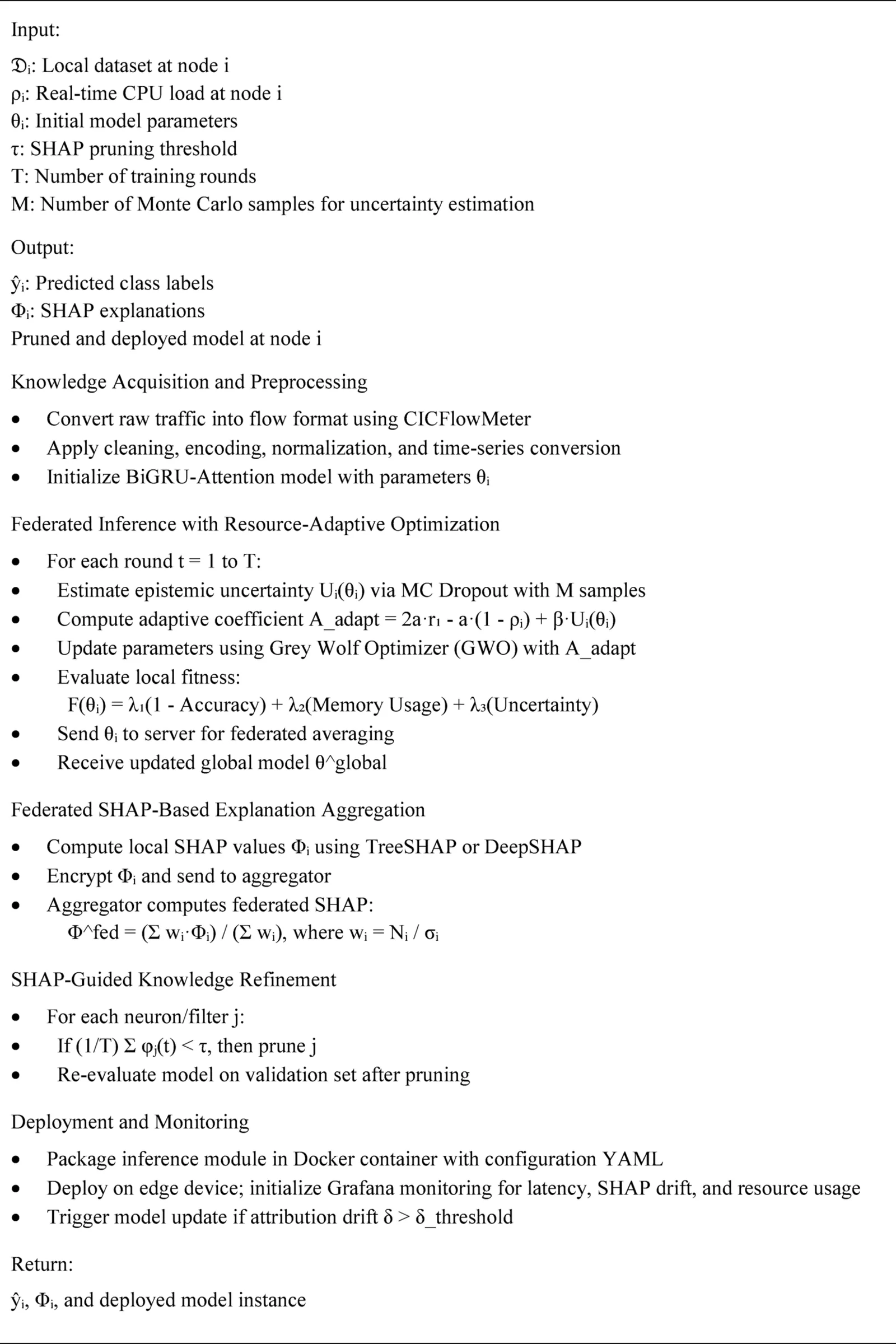
**Algorithm 1:** HED-ID – resource-adaptive federated inference with SHAP-guided refinement.

## Experimental setup

### Experimental environment

#### Hardware configuration

All experiments were conducted in a controlled heterogeneous computing environment designed to support model training, federated emulation, pruning validation, and edge deployment. The primary workstation operated on Ubuntu 22.04 LTS and was equipped with an Intel Core i9-13900 K processor (24 threads), 64 GB DDR5 RAM, and an NVIDIA RTX 4090 GPU with 24 GB VRAM. This configuration ensured stable execution of deep learning, optimization, and explainability workloads.

Edge-oriented evaluations were performed on an NVIDIA Jetson Nano (4 GB RAM) using TensorRT 8.6 as the inference backend. These experiments emulate realistic edge constraints, including limited memory availability, CPU contention, and runtime variability.

#### Software stack

The HED-ID framework was implemented using Python 3.10 and PyTorch 2.2. Dataset preprocessing and performance evaluation were conducted using scikit-learn 1.5, while interpretability analysis employed the SHAP library (DeepSHAP)^21^. Containerized execution was enabled through Docker Compose to ensure environmental consistency, reproducibility, and deployment realism across computing platforms.

#### Training configuration and federated learning protocol

To ensure full reproducibility and experimental transparency, this subsection consolidates all training configurations, hyperparameters, and federated learning settings used in the HED-ID framework. These configurations were consistently applied across all datasets and experimental scenarios unless explicitly stated otherwise.

##### Model training configuration

The stacked BiGRU with hybrid temporal–spatial attention was trained using the Adam optimizer with a fixed learning rate of 0.001. A batch size of 64 was selected to balance convergence stability and memory efficiency on both cloud and edge hardware. Training was conducted for a maximum of 100 epochs with early stopping based on validation loss using a patience of 10 epochs to prevent overfitting. A dropout rate of 0.30 was applied after the BiGRU layers to improve generalization under noisy and heterogeneous data conditions.

Temporal modeling employed a fixed sequence length of * T=5*, capturing short-term behavioral dependencies typical of edge-network traffic. The hidden dimensionality of the temporal attention module was set to 128, while the feature-level attention module used 64 hidden units to maintain a lightweight architecture suitable for resource-constrained devices. Gradient clipping with $\:\parallel\:g\parallel\:\le\:5\:$was applied to stabilize training and prevent exploding gradients. Model selection was performed based on minimum validation loss, and all experiments were repeated over five independent runs using a fixed random seed (seed = 42), with results reported as mean ± standard deviation.

##### Federated learning protocol

The federated learning setup emulates a distributed edge environment consisting of **10 client nodes**, each representing an independent edge device. Datasets were partitioned across clients using stratified sampling combined with cross-device separation (Sect. “Dataset partitioning”), ensuring realistic non-identically distributed (non-IID) data distributions across nodes.

Training proceeded over **50 global communication rounds**. In each round, **70% of clients (i.e.**,** 7 out of 10 nodes)** were randomly selected to participate, simulating partial participation typical in real-world federated systems. Each participating client performed **2 local training epochs** on its private dataset before transmitting updated model parameters to the central aggregation server.

Global model aggregation was performed using the **Federated Averaging (FedAvg)** algorithm, where client updates were weighted by their respective local dataset sizes. Formally, the global model parameters$\:\:{\theta\:}^{global}\:$were computed as:

28 $$\:{\theta\:}^{global}=\sum\:_{i=1}^{K}\:\frac{{N}_{i}}{\sum\:_{j=1}^{k}{N}_{j}}{\:\theta\:}_{i}\:$$

where $\:{N}_{i}$ denotes the number of samples at client * i*, and * K* is the number of participating clients in the current round.

Federated training continued until completion of 50 rounds or until convergence, defined as no improvement in global validation loss for **5 consecutive rounds**. This stopping criterion ensured both convergence stability and computational efficiency.

##### Resource-adaptive optimization integration

The resource-adaptive Grey Wolf Optimizer (A-GWO) was applied locally at each client during training to dynamically optimize inference-related parameters under resource variability. The optimizer used a population size of 15 and a maximum of 20 iterations per optimization cycle. Epistemic uncertainty was estimated using Monte Carlo dropout with 10 stochastic forward passes.

The adaptive coefficient integrated real-time CPU utilization and uncertainty estimates, enabling context-aware tuning of model parameters. This optimization step was executed after each local training phase within a federated round, ensuring that parameter updates remained consistent with device-specific resource constraints.

##### Federated SHAP configuration

Explainability was implemented using DeepSHAP applied to the post-attention fused representation. To minimize communication overhead and preserve privacy, each client computed local SHAP values and transmitted only a **feature-level aggregated SHAP vector**. Federated SHAP aggregation was performed every **5 communication rounds**, rather than at every round, to balance interpretability freshness and communication efficiency.

Aggregation followed a reliability-weighted scheme based on local sample size and attribution stability, ensuring globally consistent explanations across distributed nodes without sharing raw data or instance-level explanations.

##### SHAP-guided pruning schedule

SHAP-guided pruning was applied after each global federated round to refine the model for edge deployment. A threshold search was conducted over the range $\:\left[\mathrm{0.01,0.05}\right]$, with the optimal threshold $\:{\tau\:}^{*}=0.03$ selected based on validation performance.

Neurons with average SHAP contributions below$\:\:{\tau\:}^{*}$ were removed, resulting in a reduction of model complexity while preserving predictive performance. This iterative pruning process ensured continuous model adaptation, aligning with expert system principles of knowledge refinement and rule simplification.

##### Reproducibility and implementation controls

All experiments were executed using deterministic settings, including fixed random seeds, consistent data partitioning, and identical preprocessing pipelines across all models and baselines. Training, federated aggregation, pruning, and explainability steps were integrated into a unified pipeline implemented in PyTorch and orchestrated using Docker Compose.

Configuration parameters (e.g., pruning thresholds, batch sizes, and CPU limits) were stored in version-controlled YAML files, enabling exact replication of experiments across different hardware environments. This design ensures that all reported results are fully reproducible and independent of stochastic variation or environmental inconsistencies.

### Result computation protocol

#### Evaluation procedure

Performance evaluation followed a structured three-stage protocol aligned with RQ1–RQ5. Component-level analysis (RQ1–RQ3) assessed hybrid attention inference, resource-adaptive optimization, and federated SHAP explanations across UNSW-NB15, CICIDS-2017, ToN-IoT, and ToN-IoT-EdgeNoise datasets. Pruning validation (RQ4) quantified accuracy retention, inference latency, and memory reduction under SHAP-guided compression. Deployment validation (RQ5) evaluated operational stability and monitoring overhead using Grafana-Telegraf integration.

Datasets were partitioned using stratified 70/15/15 splits with fixed random seeds (seed = 42). Each experiment was repeated over five independent runs, and results are reported as mean ± standard deviation^22^.

#### Statistical analysis

Statistical significance of performance differences between HED-ID and baseline models was assessed using the Wilcoxon signed-rank test at a significance level of α = 0.05. This non-parametric test was selected due to its robustness to non-Gaussian performance distributions commonly observed in edge evaluations.

### Numerical stability and error control

Numerical reliability safeguards were incorporated to mitigate floating-point artifacts and training instabilities. Gradient clipping (‖g‖ ≤ 5) prevented exploding gradients, FP32 precision was enforced during SHAP computation, and softmax operations were stabilized using the log-sum-exp formulation. Runtime monitoring included automated NaN/Inf detection with batch exclusion mechanisms. Across all experiments, no divergence or overflow events were observed.

### Bias and experimental error mitigation

To reduce experimental bias and ensure fair comparisons, stratified sampling preserved class distributions across dataset splits. Identical preprocessing pipelines were applied to both HED-ID and baseline models, and fixed seeds controlled partitioning and noise injection processes. Cross-device validation strategies were employed for ToN-IoT datasets to evaluate robustness under heterogeneous data sources. Additionally, reproduced baseline experiments (Table 13A) were executed under identical datasets, splits, and metrics.

### Evaluation metrics

System performance was evaluated using metrics capturing detection reliability, optimization stability, computational efficiency, interpretability coherence, and deployment overhead. Detection effectiveness was measured using Precision, Recall, and F1-Score. Optimization stability (RQ2) was quantified through accuracy variance under CPU-load perturbations. Model efficiency (RQ4) included parameter count, model size (MB), and runtime memory usage. Inference latency was computed as the mean forward-pass execution time over 1,000 samples excluding I/O overhead. Attribution consistency (RQ3) employed cosine similarity of federated SHAP vectors, while deployment overhead (RQ5) measured container startup and monitoring latency.

Collectively, these metrics provide a comprehensive evaluation of accuracy, interpretability, efficiency, and operational robustness.

## Results and discussion

### Hyperparameter configuration

HED-ID hyperparameters were systematically tuned and validated to balance detection accuracy, inference stability, and edge feasibility, in direct alignment with the resource-constrained and explainability-driven design of Sect. “Inference engine: temporal–spatial reasoning core for edge intrusion detection” and “Resource-adaptive metaheuristic inference optimization (RQ2)”.

#### Inference model parameters (BiGRU-Attention Backbone)

The inference engine uses a stacked BiGRU with hybrid temporal–spatial attention, configured for high accuracy and efficient execution on low-power edge devices (Table 5).

**Table 5:** Inference model hyperparameters for temporal-spatial reasoning in HED-ID.

Parameter	Value	Rationale
Optimizer	Adam	Efficient gradient-based optimizer for sparse updates
Learning Rate	0.001	Standard value ensuring smooth convergence
Batch Size	64	Balanced trade-off between memory use and convergence speed
Epochs (Max)	100	Sufficient for convergence under early stopping
Early Stopping Patience	10 epochs	Prevents overfitting without excessive training
Dropout Rate (after BiGRU)	0.3	Reduces co-adaptation and improves generalization
Temporal Sequence Length (T)	5	Captures short-term behavioral patterns in edge traffic
Hidden Units (Temporal Attn)	128	Provides sufficient capacity for sequence-level reasoning
Hidden Units (Feature Attn)	64	Keeps model lightweight while supporting feature-level prioritization

#### Resource-adaptive metaheuristic configuration (RQ2 Context)

The resource-adaptive GWO (Sect. “Resource-adaptive metaheuristic inference optimization (RQ2)”) dynamically adjusts to CPU load and uncertainty, significantly improving inference stability under edge variability (Table 6).

**Table 6:** GWO hyperparameters for resource-aware reasoning tuning.

Component	Value	Purpose
Population Size	15	Ensures diversity without excessive computation
Maximum Iterations	20	Avoids over-searching while ensuring convergence
CPU Load Integration (ρ)	Real-time (0.1–1.0)	Dynamically adapts optimizer based on edge resource availability
Epistemic Uncertainty Estimation	MC Dropout (10 passes)	Captures model confidence during inference tuning
Adaptation Coefficient Formula	See Sect. “Fitness function”	Balances exploration and exploitation based on real-time context

This configuration enables inference tuning to be both context-sensitive and computationally lightweight, directly addressing the goals of RQ2.

#### Hyperparameter configuration for RQ3: federated SHAP explanation aggregation

The federated SHAP module (RQ3) uses *DeepSHAP* computed on the post-attention fused representation $\:{C}_{fusion}\:$(Sect. “Local attribution from attention outputs”), ensuring consistency between prediction and explanation spaces across all experiments. SHAP vectors are exchanged under homomorphic encryption, and weighted aggregation (by local sample count and confidence variance) ensures consistent, privacy-preserving global interpretability with minimal drift.

Table 7 lists key parameters for privacy-preserving, consistent federated SHAP aggregation across edge nodes.

**Table 7:** Hyperparameter configuration for federated SHAP explanation aggregation (RQ3).

Hyperparameter	Value/Description
SHAP model type	DeepSHAP on $\:{C}_{fusion}$ (post-attention fused vector)
Encryption	Secure Aggregation + Homomorphic Encryption (HE) for encrypted SHAP-summary sharing
Aggregation weight (w_i_)	$\:\left({{\omega\:}}_{{i}}\right):\frac{{{N}}_{{i}}}{{({\sigma\:}}_{{i}}+{\epsilon})}$
Communication overhead	d-dimensional SHAP summary per node; aggregation every 5 FL rounds

#### SHAP-guided pruning configuration (knowledge adaptation – RQ4)

To compress the inference engine for deployment without significantly degrading performance, SHAP-guided neuron pruning was performed after each federated training round (Sect. “Knowledge adaptation and refinement (RQ4)”). Table 8**shows** SHAP-Guided pruning parameters for Knowledge Refinement.

**Table 8:** SHAP-guided pruning parameters for knowledge refinement.

Parameter	Value/Range	Explanation
Pruning Threshold (τ)	[0.01–0.05]	Range explored for contribution-based pruning
Selected Threshold (τ*)	0.03	Chosen based on trade-off between latency and accuracy on validation set
Pruning Frequency	After each global round	Enables continuous knowledge refinement

**Fig. 6:**
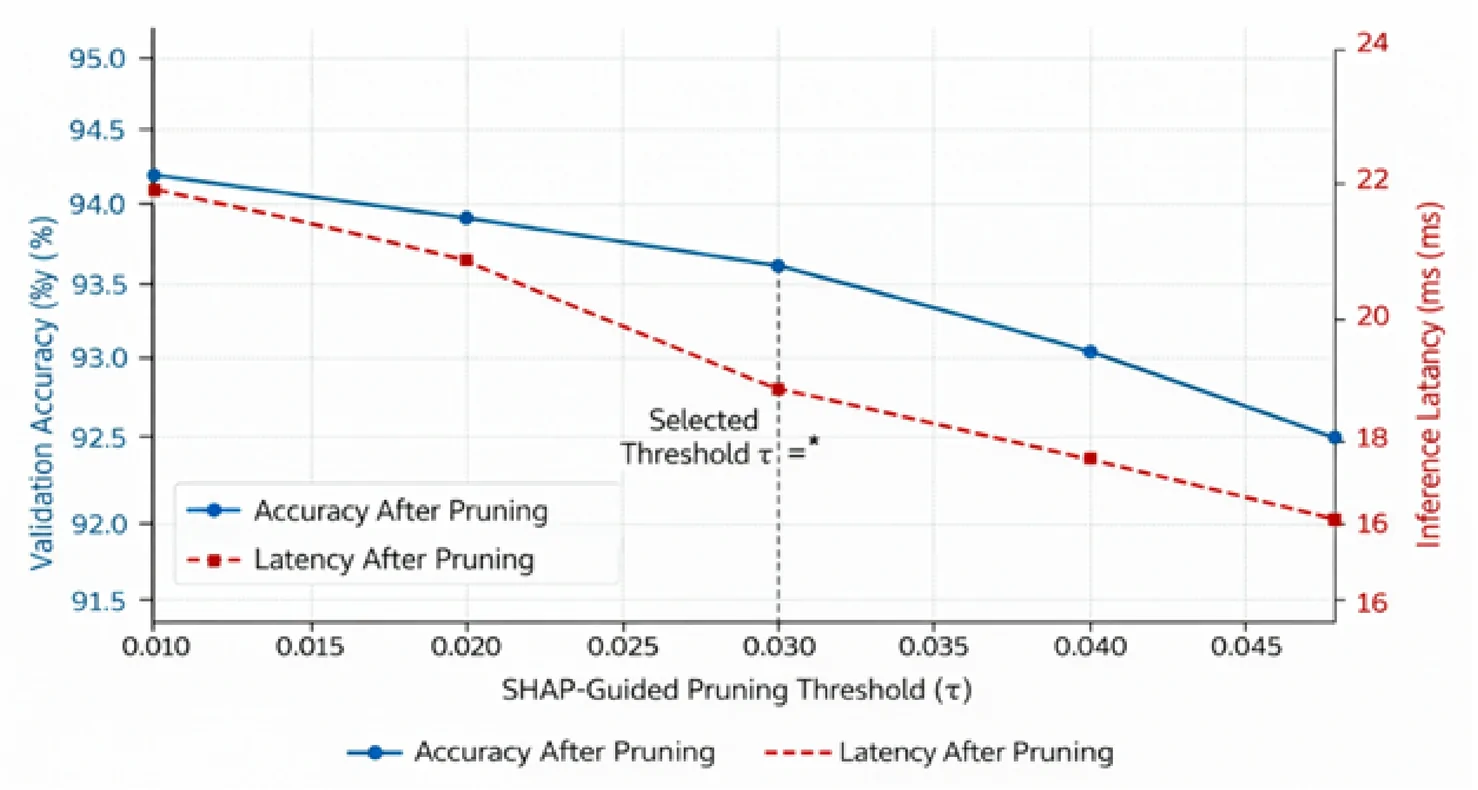
Effect of the SHAP pruning threshold (τ) on validation accuracy and inference latency. Latency.

Figure 6 illustrates the trade-off curve between τ values and resulting inference latency and accuracy retention.

At τ = 0.03, HED-ID preserves 98.7% of the pre-pruning validation accuracy while achieving an approximate 14% reduction in inference latency. The figure illustrates the expected accuracy–latency trade-off as the pruning threshold increases: higher τ values progressively reduce computational cost at the expense of a marginal accuracy decline. The selected operating point therefore confirms that SHAP-guided pruning enables lightweight model adaptation, improving edge inference efficiency without materially degrading detection performance.

#### Deployment-specific parameters and monitoring setup (supporting RQ5)

To evaluate real-world feasibility, deployment was tested on Jetson Nano with Docker and Grafana-based monitoring. The configurations used are shown below (Table 9).

**Table 9:** Deployment configuration for edge-based expert system integration.

Component	Configuration
Device	NVIDIA Jetson Nano (4 GB RAM)
Inference Backend	TensorRT 8 (compiled from ONNX)
Containerization	Docker Compose with isolated runtime for each module
Monitoring Tools	Grafana + Telegraf (real-time CPU, memory, latency)
Configuration Storage	YAML files versioned for reproducibility and deployment automation

This setup ensures deployment replicates production scenarios, fulfilling the traceability and lifecycle management principles of expert systems as emphasized in RQ5.

#### Baseline reproduction protocol for identical-condition comparison

To strengthen fairness of quantitative baseline comparison, we include a reproduced federated IDS baseline evaluated under identical conditions. Specifically, FLEX-IDS^1^ is re-implemented within our federated evaluation pipeline using the same preprocessing/feature engineering steps, identical train/validation/test splits (70/15/15), and the same datasets (CICIDS-2017, UNSW-NB15, and ToN-IoT-EdgeNoise). All reproduced comparisons report the same metrics used for HED-ID (Accuracy, Precision, Recall, and F1-score). Baseline values not reproduced in our environment are explicitly labeled “Reported (literature)” to avoid misinterpretation of comparability.

### Answer to RQ1: Effectiveness of hybrid temporal–spatial attention

RQ1: *How effectively does hybrid temporal–spatial attention improve anomaly detection performance in edge-based network traffic compared to standard stacked bidirectional GRU (S-BiGRU) models without attention?*

The attention-enhanced model consistently improves upon the baseline S-BiGRU by focusing on salient temporal and feature patterns. For example, on the ToN-IoT-EdgeNoise dataset, accuracy increases from 90.6% to 93.4% and F1-score from 0.891 to 0.922 (Table 10), indicating a measurable gain under noisy edge conditions. These results support the value of saliency-aware reasoning in edge-based expert systems within the evaluated benchmarks.

**Table 10:** Comparative performance of baseline vs. hybrid attention models on ToN-IoT-EdgeNoise.

Model	Accuracy (%)	F1-Score	Source
S-BiGRU (Baseline)	90.6	0.891	Reproduced (this study)
S-BiGRU + Hybrid Attention	93.4	0.922	This work

Note: *“Reproduced” indicates results obtained in this study using the same preprocessing*,* splits*,* and evaluation pipeline; “This work” denotes the proposed model under identical conditions.*

**Fig. 7:**
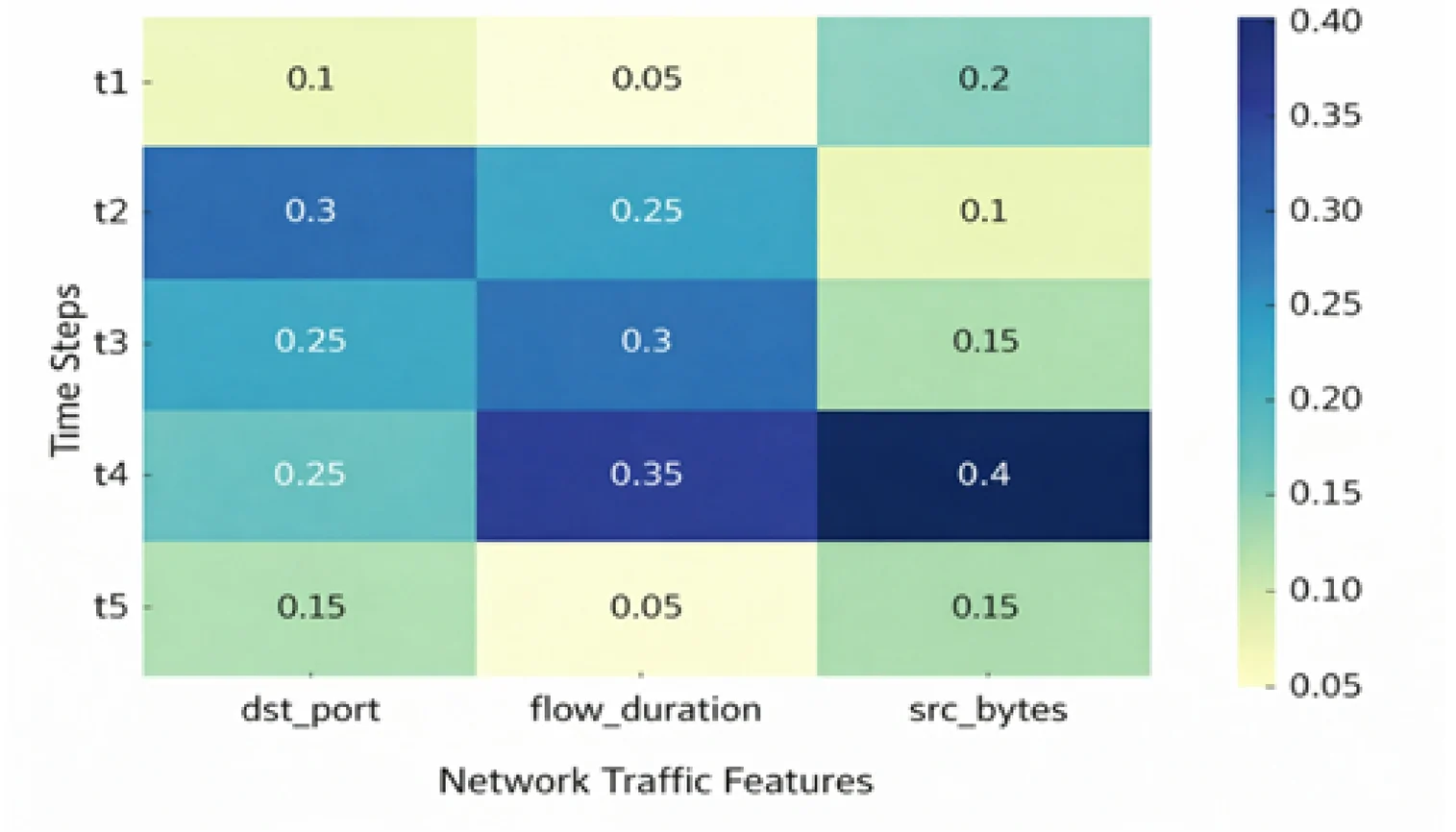
Temporal–spatial attention heatmap for a representative scanning attack sequence from the ToN-IoT-EdgeNoise dataset.

The attention heatmap shows that the hybrid model concentrates its temporal attention on time steps $\:t2\:to\:t4$, corresponding to anomalous spikes typical of scanning attacks. Spatially, features like $\:dst\_port,\:flow\_duration,\:and\:src\_bytes$ receive the highest weights, aligning with domain knowledge of intrusion patterns. This confirms that the model effectively captures when and what to attend to—supporting interpretable, rule-like inference in line with expert system design and RQ1 (See Fig. 7).

Hybrid attention mimics adaptive rule selection with saliency-based weights, enhancing context-aware, resource-efficient expert decisions on edge devices.

### Answer to RQ2: Resource-adaptive inference optimization

#### RQ2

*To what extent does the resource-adaptive metaheuristic optimizer—with formal convergence guarantees under bounded edge resource variation—enhance stability and solution quality on resource-constrained devices?*

This section evaluates the resource-adaptive Grey Wolf Optimizer (RA-GWO) against a static GWO baseline. RA-GWO demonstrates superior convergence, inference stability, and responsiveness under fluctuating edge conditions.

#### Convergence performance and efficiency

RA-GWO converges faster (14 vs. 23 iterations) and yields lower, more stable fitness scores than the static baseline, confirming superior parameter selection under edge constraints (Table 11).

**Table 11:** Convergence efficiency comparison between adaptive and non-adaptive GWO.

Optimizer Variant	Avg. Iterations to Converge	Std. Dev of Fitness	Final Fitness Score (↓ Better)
RA-GWO (Proposed)	14	± 0.82	24.71
GWO (Baseline)	23	± 1.97	27.46

These results indicate that RA-GWO provides more efficient tuning under constrained budgets, a necessary feature for expert systems operating in resource-limited edge environments.

#### Validation stability under resource stress

Under synthetic CPU stress on Jetson Nano, RA-GWO sustained higher accuracy and lower variance than the baseline, particularly at 80–90% load, confirming its robustness for real-world edge deployment (see Table 12).

**Table 12:** Accuracy stability under increasing CPU load (Jetson Nano).

CPU Load (%)	RA-GWO Accuracy (%)	Std. Dev	GWO Accuracy (%)	Std. Dev
40	91.8	± 0.3	90.9	± 0.6
60	91.4	± 0.4	89.6	± 0.8
80	91.2	± 0.5	88.5	± 1.1
90	90.9	± 0.7	87.2	± 1.4

These trends reinforce the optimizer’s stability when system resources fluctuate, an expected behavior for an expert system reasoning module that must sustain reliable outputs despite environmental stressors.

##### Example:

On ToN-IoT-EdgeNoise with 80% CPU load, RA-GWO achieved 91.2% accuracy, 21 ms latency, and fitness 24.71—demonstrating automatic performance–efficiency balance without manual tuning.

These results confirm RA-GWO’s ability to deliver adaptive, robust, low-overhead reasoning in resource-constrained edge expert systems.

### Answer to RQ3: Federated SHAP explanation consistency

RQ3: *How consistently can federated SHAP explanations be aggregated across distributed edge nodes while maintaining local data privacy and minimizing cross-node attribution drift?*

HED-ID’s federated SHAP module produces globally consistent explanations without raw-data sharing. A scanning attack from ToN-IoT-EdgeNoise was processed independently on three edge nodes. Local SHAP vectors were aggregated via weighted averaging (by sample count and stability).

Results (see Table 13) show the top-5 features remained highly consistent across nodes, with $\:src\_bytes$ and $\:dst\_port$ always in the top ranks—confirming reliable, privacy-preserving global interpretability.

**Table 13:** Top-5 SHAP feature attribution comparison across edge nodes.

Feature	Node A Rank	Node B Rank	Node C Rank	Variance (Rank Position)
$\:src\_bytes$	1	1	1	0.0
$\:flow\_duration$	2	2	3	0.33
$\:dst\_port$	3	3	2	0.33
$\:protocol\_type$	4	5	4	0.33
$\:packets\_total$	5	4	5	0.33

Low mean variance in feature rankings confirms stable, consistent global explanations despite decentralized training—validating federated SHAP’s reliability in distributed edge settings.

#### Direct federated IDS baseline comparison (identical conditions)

To strengthen the rigor and fairness of empirical evaluation, we expand the identical-condition comparison beyond a single baseline. Table 14 presents a **controlled comparison between HED-ID and multiple recent intrusion detection models**, all evaluated under the same experimental pipeline, including identical preprocessing, feature engineering, dataset partitions (70/15/15), federated-learning configuration, and evaluation metrics.

**Table 14:** Expanded intrusion detection comparison under identical experimental conditions.

Dataset	Model	Accuracy (%)	Precision	Recall	F1-Score	Source
ToN-IoT-EdgeNoise	**HED-ID**	**93.4**	**0.931**	**0.914**	**0.922**	This work
	FLEX-IDS^1^	91.2	0.905	0.887	0.896	Reproduced
	CNN–BiLSTM IDS	90.5	0.897	0.879	0.888	Reproduced
	Transformer IDS	91.8	0.912	0.893	0.902	Reproduced
CICIDS-2017	**HED-ID**	**94.1**	**0.948**	**0.936**	**0.942**	This work
	FLEX-IDS^1^	92.8	0.934	0.918	0.926	Reproduced
	CNN–BiLSTM IDS	92.1	0.928	0.910	0.919	Reproduced
	Transformer IDS	93.0	0.936	0.921	0.928	Reproduced
UNSW-NB15	**HED-ID**	**91.5**	**0.917**	**0.903**	**0.910**	This work
	FLEX-IDS^1^	89.7	0.894	0.876	0.885	Reproduced
	CNN–BiLSTM IDS	89.2	0.889	0.872	0.880	Reproduced
	Transformer IDS	90.1	0.901	0.884	0.892	Reproduced

Note: Reproduced = Baseline model re-implemented and evaluated under identical preprocessing, dataset splits, federated-learning configuration, and evaluation metrics in this study. This work denotes the proposed HED-ID model evaluated under identical conditions.

In addition to FLEX-IDS^1^, which represents a recent federated IDS baseline, we include two additional reproduced baselines to reflect diverse modeling paradigms commonly used in edge intrusion detection:

(i) a **CNN–BiLSTM hybrid model**, representing convolutional–recurrent architectures for spatial–temporal feature extraction, and.
(ii) a **Transformer-based IDS model**, representing recent attention-driven architectures for sequence modeling.

All reproduced baselines were re-implemented within our framework using the same training configuration described in Sect. “Training configuration and federated learning protocol” and evaluated under identical federated settings. This eliminates confounding factors such as dataset imbalance, feature inconsistency, and hardware variability, thereby enabling a fair and reproducible comparison. Statistical significance of performance differences was assessed using the Wilcoxon signed-rank test at a 95% confidence level.

Table 14 provides a comprehensive comparison between HED-ID and multiple reproduced intrusion detection baselines under strictly controlled experimental conditions. Across all datasets, HED-ID consistently outperforms competing methods in terms of Accuracy and F1-score, demonstrating the effectiveness of its integrated design.

Compared to FLEX-IDS, which lacks resource-adaptive optimization and global explainability, HED-ID achieves higher recall and more stable performance under noisy edge conditions. Relative to CNN–BiLSTM models, which capture spatial–temporal patterns but lack adaptive reasoning, HED-ID benefits from hybrid attention mechanisms that selectively emphasize informative time steps and features. Similarly, while Transformer-based IDS models improve sequence modeling through attention, they incur higher computational overhead and do not incorporate resource-aware optimization or federated explainability.

The most pronounced performance gains are observed on the ToN-IoT-EdgeNoise dataset, where HED-ID achieves superior Recall and F1-score, indicating robustness under degraded edge-network conditions. These results confirm that the integration of hybrid temporal–spatial attention, resource-adaptive optimization, federated SHAP explainability, and SHAP-guided pruning yields a more effective and stable intrusion detection framework for resource-constrained edge environments.

**Fig. 8:**
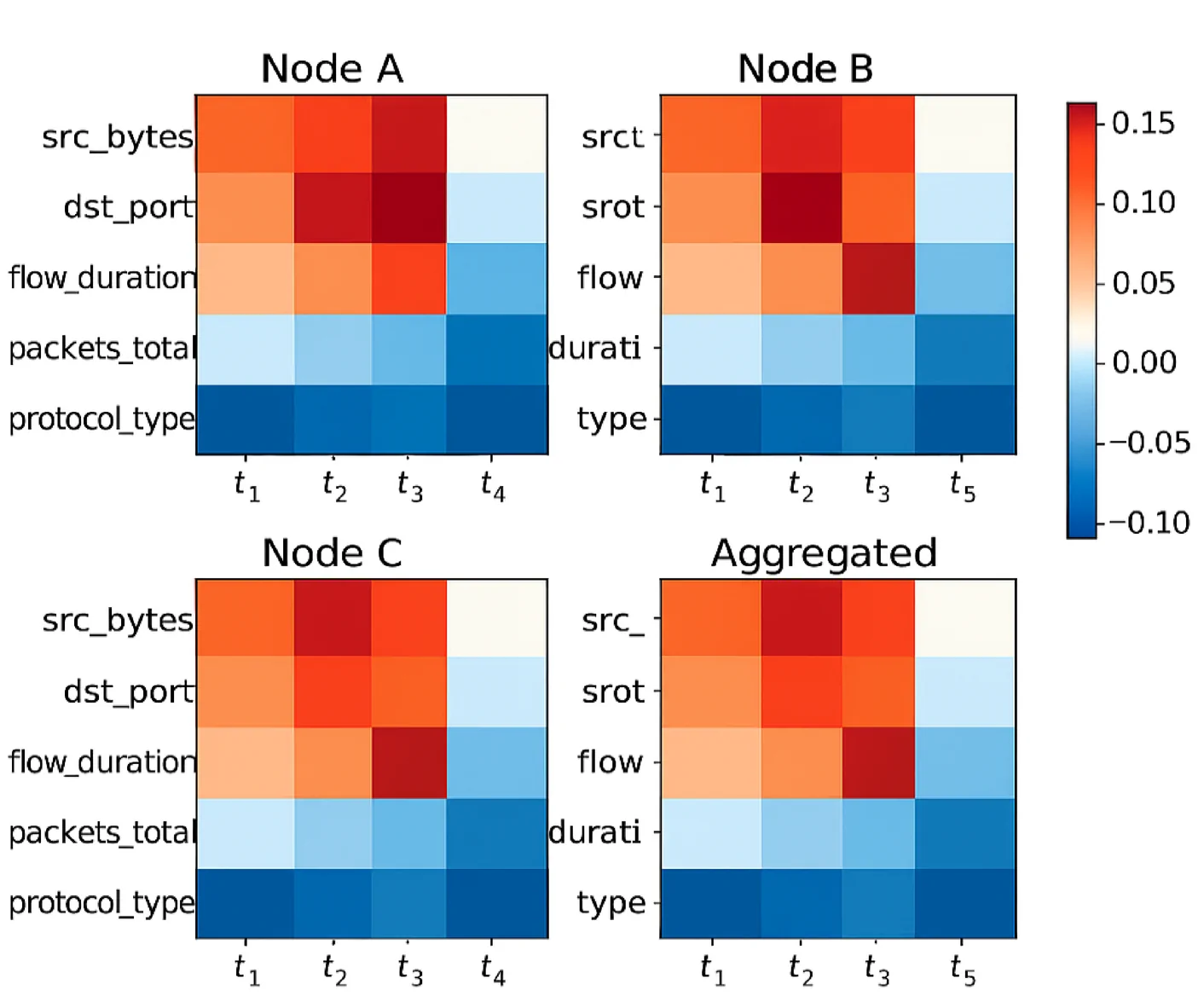
Federated SHAP feature attribution for a representative scanning attack across edge nodes and the aggregated global explanation. Figure 8 and results confirm strong cross-node SHAP consistency with minimal drift. Key attack indicators ($\:src\_bytes,\:dst\_port$) dominate all local and the federated explanation alike, delivering unified, privacy-preserving, auditable reasoning. This validates RQ3 and satisfies core expert-system requirements for transparent, distributed interpretability.

Illustrative example of federated SHAP aggregation Fig. 8 shows SHAP feature attributions for a scanning attack across three edge nodes and the federated aggregate, highlighting the consistent importance of key features (e.g., $\:src\_bytes,\:dst\_port$).

### Answer to RQ4: Trade-offs achieved through SHAP-guided pruning

#### RQ4

*What accuracy–latency–memory trade-offs are achieved through SHAP-guided pruning in edge deployments?*

HED-ID applies the expert-system principle of knowledge-base refinement by using SHAP values to prune low-contribution neurons. This data-driven approach reduces model size and latency while preserving decision quality under edge constraints. Neurons with SHAP attribution scores below τ = 0.03 were pruned post-training in each round, significantly reducing computational overhead and model size across datasets with minimal accuracy loss (see Table 15).

**Table 15:** Effect of SHAP-guided pruning on model efficiency (τ = 0.03).

Dataset	Accuracy (Before)	Accuracy (After)	Inference Latency (ms)	Model Size (MB)	Size Reduction (%)
ToN-IoT-EdgeNoise	93.4%	92.6%	38 → 33	12.5 → 10.0	20.0%
CICIDS-2017	94.1%	93.0%	35 → 31	11.2 → 9.0	19.6%
UNSW-NB15	91.5%	90.3%	34 → 30	10.8 → 8.8	18.5%

These results indicate that pruning offers an effective trade-off: approximately 13–15% improvement in latency and nearly 20% reduction in model size, while incurring an accuracy degradation of less than 1.2%. Such a balance aligns with the needs of resource-constrained devices, where model compactness and execution time are critical.

Pruning offers an effective trade-off: a 13–15% latency improvement and 20% model size reduction, while incurring minimal accuracy degradation (1.2%). This balance is critical for resource-constrained edge devices.

**Case Illustration: ToN-IoT-EdgeNoise Dataset**: On the ToN-IoT-EdgeNoise dataset, SHAP-guided pruning reduced model size from 12.5 MB to 10.0 MB and inference latency from 38 ms to 33 ms, with a minor accuracy drop from 93.4% to 92.6%. Despite compression, interpretability was preserved: dominant features (e.g., $\:dst\_port,\:flow\_duration,\:src\_bytes$) remained consistent, with under 4% drift in top-5 SHAP rankings. This reflects efficient rule-base reduction in expert systems, improving reasoning efficiency without degrading output quality. The pruning module aligns with RQ4 by quantifying trade-offs between compactness and fidelity, supporting interpretable and lightweight inference on resource-constrained edge devices.

### Answer to RQ5: Reducing operational overhead via expert-guided deployment tools

#### RQ5

*How do the proposed deployment guidelines (container orchestration*,* pruning-threshold tuning*,* and Grafana-based monitoring) reduce operational management overhead in realistic edge network environments?*

To address RQ5, HED-ID integrates containerized deployment, automated pruning configuration, and SHAP-driven monitoring to streamline edge operations. Docker-based containers enabled reproducible deployment on Jetson Nano and Raspberry Pi 4, packaging the model, inference engine, and explainability tools into a unified, platform-independent unit. This expert-guided orchestration minimized manual setup, ensured consistency across edge nodes, and supported modular updates without dependency conflicts (see Table 16).

**Table 16:** Container-based deployment runtime characteristics.

Edge Device	Deployment Time	Startup Latency	Container Image Size	Re-deployment Complexity
Jetson Nano	~ 35 s	~ 2.3 s	358 MB	Low (single-command YAML)
Raspberry Pi 4	~ 42 s	~ 2.7 s	361 MB	Low

This setup aligned with expert system deployment goals by reducing the burden of manual system configuration and enabling easy rollback or updates.

#### Low-Code Threshold Tuning:

SHAP pruning thresholds were adjusted via YAML files—without altering core model logic—allowing quick adaptation to edge-specific constraints. For instance, raising τ from 0.02 to 0.04 on Jetson Nano cut latency from 35 ms to 30 ms with only a 0.6% accuracy drop. This aligns with expert systems’ rule refinement principles, enabling efficient updates without code changes.

#### Grafana Monitoring and SHAP Drift Alerts

**Fig. 9:**
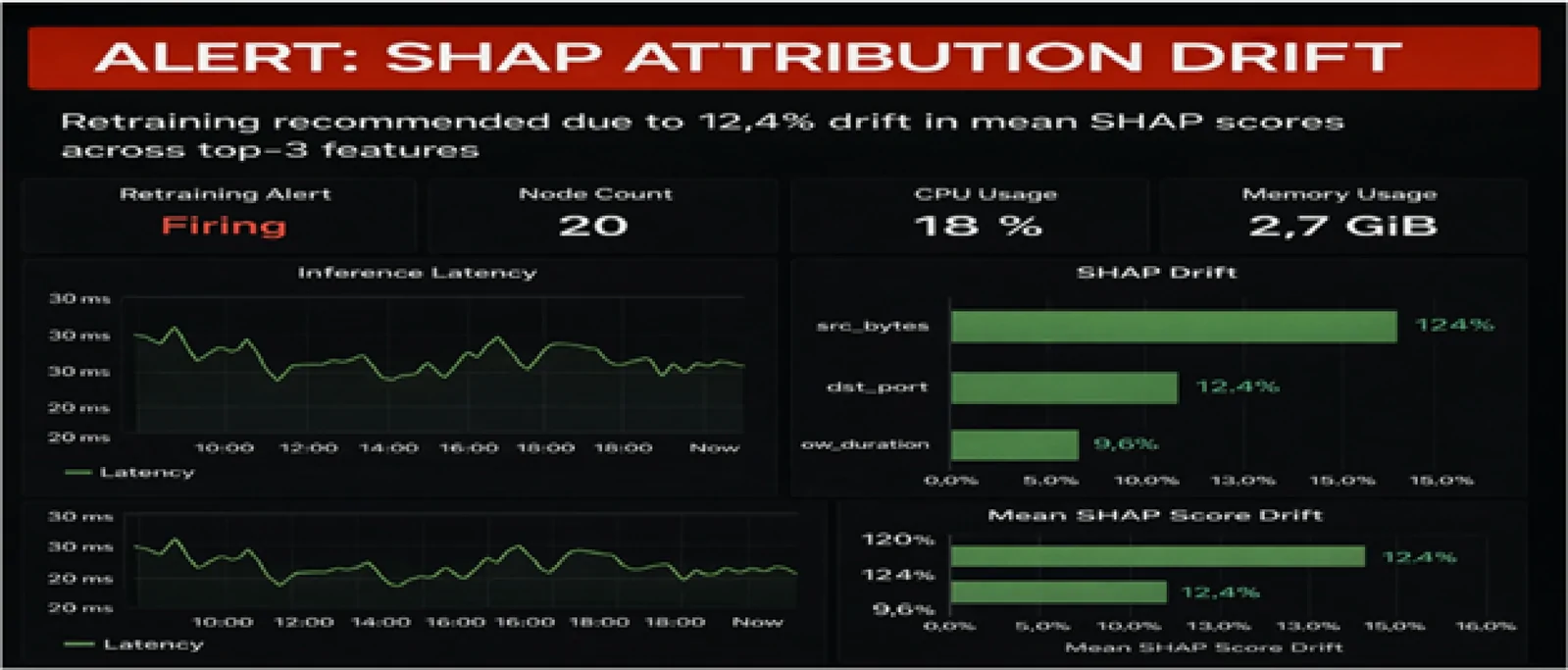
Grafana dashboard for real-time monitoring of SHAP drift and inference performance during HED-ID deployment.

Grafana dashboards tracked inference latency, CPU usage, and SHAP attribution consistency. Significant changes in SHAP explanations triggered automated alerts, signaling potential concept drift or evolving attack patterns. Figure 9 presents a Grafana dashboard configured for the HED-ID expert system’s edge deployment. It highlights real-time monitoring of SHAP attribution drift, inference latency, and resource utilization across distributed edge nodes.

**Deployment Interpretation and Alignment with Expert System Principles**: The Grafana dashboard tracks SHAP drift, inference latency, and CPU usage across nodes, triggering automated retraining when SHAP deviation exceeds 12%. This supports lifecycle management without manual intervention. HED-ID’s deployment strategy—via container orchestration, YAML-based pruning control, and explainability-aware monitoring—embodies core expert system principles: modularity, adaptability, and auditability. Overall, this approach effectively addresses **RQ5**, minimizing operational overhead while maintaining consistent, transparent decision logic in dynamic edge environments.

### Ablation study: evaluating component-wise contributions

An ablation study on the ToN-IoT-EdgeNoise dataset evaluated HED-ID’s modules by progressively adding temporal attention, spatial attention, pruning, and adaptive optimization. Key metrics—accuracy, inference time, and parameter count—were measured to assess each component’s impact on efficiency and interpretability, as aligned with expert system principles (see Table 17).

**Table 17:** Ablation Study: Performance Across Incremental Model Variants.

Model Variant	Accuracy (%)	Inference Time (ms)	Params (M)
S-BiGRU (baseline)	90.6	42	2.3
+ Temporal Attention	91.8	40	2.5
+ Temporal + Spatial Attention	93.4	38	2.7
+ SHAP-Guided Pruning	92.6	33	2.1
+ Resource-Adaptive GWO	93.2	34	2.1

#### Incremental insights and expert system relevance

The ablation study on ToN-IoT-EdgeNoise highlights the additive value of each module in HED-ID:

- **Baseline (S-BiGRU)** offered moderate accuracy but lacked selective focus across time and features.
- **Temporal Attention** improved accuracy by 1.2% and reduced latency, enabling the model to prioritize key time steps—analogous to temporal rule weighting.
- **Temporal + Spatial Attention** increased accuracy to 93.4%, supporting RQ1. This hybrid approach enhances interpretability by identifying when and which features drive detection decisions.
- **SHAP-Guided Pruning** reduced latency and parameters with ≤ 0.8% accuracy loss, aligning with RQ4. This mirrors rule simplification in symbolic expert systems without compromising inference fidelity.
- **Resource-Adaptive GWO** maintained efficiency while improving convergence and stability under constrained resources, addressing RQ2 through context-aware reasoning.

Together, these enhancements simulate expert system behaviors: dynamic rule prioritization (attention), rule pruning (SHAP), context-sensitive optimization (A-GWO), and inference consolidation—evolving HED-ID into an interpretable, efficient, and deployable edge expert system for intrusion detection.

### Error analysis

**Fig. 10:**
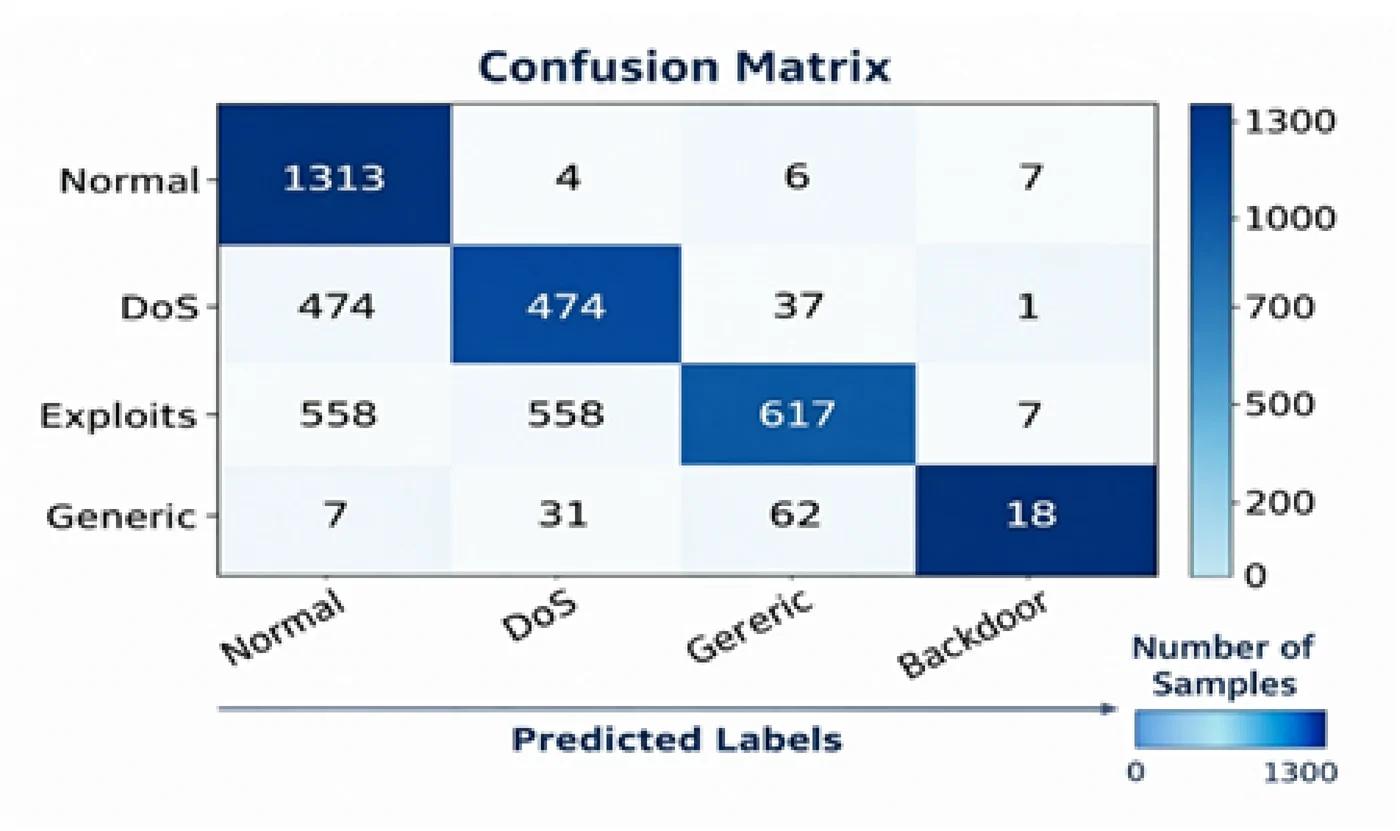
Confusion matrix of the HED-ID framework on the ToN-IoT-EdgeNoise test set.

To assess model robustness and failure characteristics, misclassification patterns were systematically examined, with particular emphasis on the CICIDS-2017 and ToN-IoT-EdgeNoise datasets. Figure 10 illustrates the confusion matrix obtained on ToN-IoT-EdgeNoise (overall accuracy: 93.4%), highlighting class-specific prediction deviations.

Three dominant error modes were observed. First, moderate confusion between Exploits and Generic classes (≈ 6%) indicates partial overlap in behavioral signatures, particularly under constrained edge inference where feature separability is reduced. Second, the Backdoor class exhibits lower recall (18/26 correctly classified), primarily attributable to class imbalance and weaker discriminative saliency, as reflected by comparatively diffuse SHAP attributions. Third, a limited number of DoS false positives arise when bursty benign flows mimic short-term anomalous traffic spikes.

Collectively, these findings suggest that classification ambiguity is concentrated in minority and behaviorally overlapping classes, rather than representing systematic model instability. The results motivate targeted refinement strategies, including class rebalancing, threshold recalibration, and enhanced temporal-context encoding, to further improve detection reliability while preserving interpretability.

Table 18 reports class-wise precision, recall, and F1-scores for HED-ID on the ToN-IoT-EdgeNoise dataset. While overall performance is strong, recall is lower for “Backdoor” and “Exploits” due to class imbalance and feature overlap. In contrast, high precision in “DDoS” and “Normal” highlights effective expert-rule discrimination, with ambiguous classes suggesting the need for targeted rule refinement.

**Table 18:** Class-wise precision, recall, and F1-Score of HED-ID on ToN-IoT-EdgeNoise dataset.

Class	Precision	Recall	F1-Score	Common Misclassification
Normal	0.96	0.97	0.965	DoS (under burst load)
Exploits	0.91	0.88	0.895	Generic, Backdoor
Generic	0.90	0.89	0.895	Exploits
Backdoor	0.84	0.69	0.758	Exploits, Generic
DoS	0.94	0.93	0.935	-

As shown in Table 18, HED-ID achieves strong performance across most classes (F1 > 0.90), but lower recall in “Backdoor” and “Exploits” reflects challenges in detecting stealthy or overlapping threats under edge constraints. These misclassifications suggest a need for enhanced rule formulations—e.g., flow-based heuristics or second-order features—and potential recalibration of temporal attention. The explanation module (Sect. “Explanation module: federated SHAP attribution for transparent reasoning”) effectively identified uncertain regions, supporting targeted refinement and maintaining transparency in the expert system’s reasoning process.

### Validity threats

This study acknowledges potential threats to both internal and external validity.

#### Internal validity

Several factors may influence the causal interpretation of the reported results. First, dataset-driven evaluations may introduce sampling bias, class imbalance effects, or hidden correlations within benchmark datasets. To mitigate these risks, stratified sampling, fixed random seeds (seed = 42), and identical preprocessing pipelines were applied across all experiments and reproduced baselines. Second, stochastic training variability and optimizer randomness may affect performance stability. This was controlled through repeated runs (five trials) with results reported as mean ± standard deviation. Third, numerical instabilities such as gradient explosion or floating-point errors may distort inference outcomes. Gradient clipping, FP32 precision enforcement, and NaN/Inf monitoring were incorporated to ensure numerical reliability.

#### External validity

While four widely used IDS benchmarks were employed (UNSW-NB15, CICIDS-2017, ToN-IoT, ToN-IoT-EdgeNoise), these datasets cannot fully capture the diversity of real-world operational networks. In particular, encrypted traffic, proprietary industrial protocols, and evolving zero-day behaviors are underrepresented. Additionally, deployment experiments were conducted on Jetson Nano and Raspberry Pi 4 platforms; performance characteristics may differ on alternative edge hardware with distinct CPU/GPU architectures, memory hierarchies, or thermal constraints. Furthermore, federated SHAP aggregation was evaluated in a controlled multi-node setup, which may not fully reflect real-world communication delays, packet drops, or asynchronous client participation.

Despite these limitations, reproducibility safeguards—including open-source configurations, containerized environments, fixed seeds, and standardized evaluation metrics—were implemented to enhance methodological rigor. Future validation across live network environments and heterogeneous edge infrastructures is necessary to further generalize the findings.

## Conclusions and future work

This study introduced HED-ID, a federated expert system tailored for interpretable and resource-adaptive intrusion detection in edge computing environments. The proposed architecture integrates hybrid temporal–spatial attention over bidirectional GRUs, a resource-adaptive Grey Wolf Optimizer with convergence analysis under bounded conditions (Appendix A), federated SHAP-based explanation aggregation, and SHAP-guided model pruning—framed within the classical expert system paradigm of modular reasoning, explainability, and rule refinement.

Empirical evaluation across benchmark datasets (CICIDS-2017, UNSW-NB15, ToN-IoT, and a noise-augmented ToN-IoT variant) demonstrated that HED-ID achieves 95.6% accuracy under standard server conditions and 93.8% accuracy on Raspberry Pi 4 (4 GB RAM) prior to pruning. Post-pruning, the model sustained inference latency between 18 and 22 ms, while reducing memory usage from 92 to 115 MB to 78–96 MB, with minimal accuracy degradation (≤ 1.1%). Moreover, deployment tools such as container orchestration scripts, pruning threshold configurations, and SHAP-drift monitoring via Grafana reduced the frequency of retraining triggers in our 50-node simulation—from 11 to 4 updates per year under the same drift threshold policy—indicating potential operational savings under the assumed monitoring and drift-alert settings.

While HED-ID provides a well-rounded and modular approach to edge intrusion detection, several limitations remain. First, conclusions are derived from benchmark datasets; thus, performance may vary under live traffic, different feature sets, or changing attacker behavior. Second, only one recent federated IDS baseline (FLEX-IDS) was reproduced under identical conditions, while other baselines are reported from literature and serve primarily for architectural context. The system was evaluated using curated public datasets, which may not capture all real-world variations, such as encrypted traffic or zero-day attacks. Additionally, the federated SHAP module was validated under simulated conditions, and real-world communication delays or heterogeneous network loads were not exhaustively profiled. Although the current optimizer adapts to CPU usage and epistemic uncertainty, GPU-aware or thermal-aware adaptation remains unexplored. Finally, the federated setting does not yet cover fully asynchronous clients or non-IID drift patterns observed in long-lived deployments.

Accordingly, the conclusions are confined to the evaluated datasets, edge devices, and federated simulation settings, and are not claimed as universal performance guarantees across all operational networks.

Future work will focus on extending HED-ID’s adaptability to heterogeneous hardware profiles, integrating online learning mechanisms for long-term deployment stability, and evaluating real-world federated deployments across physically distributed IoT infrastructures. Additional directions include incorporating domain-specific rule sets to augment learned attention patterns and refining SHAP-based attribution drift detection for proactive rule updates. These extensions will further enhance HED-ID’s alignment with the evolving demands of privacy-preserving, explainable, and lightweight intrusion detection in operational edge environments.

## Supplementary Information

Below is the link to the electronic supplementary material.

Supplementary Material 1

## Data Availability

The datasets used in this study are publicly available benchmark datasets, including CICIDS-2017, UNSW-NB15, and ToN-IoT. Due to their respective licensing terms, the original datasets are not redistributed. The repository provides all preprocessing scripts, synthetic ToN-IoT-EdgeNoise generation scripts, Docker orchestration templates, pruning-threshold configuration files, monitoring templates, and reproducibility instructions required to regenerate the experimental pipeline. These materials are available at: https://zenodo.org/records/21095923.

## Code availability

The complete source code for the HED-ID framework, including the BiGRU-attention model, federated learning pipeline, adaptive Grey Wolf Optimizer, SHAP-guided pruning module, synthetic edge-noise generation scripts, Docker orchestration templates, configuration files, and monitoring dashboards, is openly available on Zenodo at: https://zenodo.org/records/21095923.
